# Let’s go fishing: A quantitative analysis of subsistence choices with a special focus on mixed economies among small-scale societies

**DOI:** 10.1371/journal.pone.0254539

**Published:** 2021-08-04

**Authors:** Virginia Ahedo, Débora Zurro, Jorge Caro, José Manuel Galán

**Affiliations:** 1 Departamento de Ingeniería de Organización, Escuela Politécnica Superior, Universidad de Burgos, Burgos, Spain; 2 Departamento de Arqueología y Antropología, HUMANE – Human Ecology and Archaeology, Institución Milá y Fontanals de Investigación en Humanidades – Consejo Superior de Investigaciones Científicas (CSIC), Barcelona, Spain; Universidade Federal de Pernambuco, BRAZIL

## Abstract

The transition to agriculture is regarded as a major turning point in human history. In the present contribution we propose to look at it through the lens of ethnographic data by means of a machine learning approach. More specifically, we analyse both the subsistence economies and the socioecological context of 1290 societies documented in the Ethnographic Atlas with a threefold purpose: (i) to better understand the variability and success of human economic choices; (ii) to assess the role of environmental settings in the configuration of the different subsistence economies; and (iii) to examine the relevance of fishing in the development of viable alternatives to cultivation. All data were extracted from the publicly available cross-cultural database D-PLACE. Our results suggest that not all subsistence combinations are viable, existing just a subset of successful economic choices that appear recurrently in specific ecological systems. The subsistence economies identified are classified as either primary or mixed economies in accordance with an information-entropy-based quantitative criterion that determines their degree of diversification. Remarkably, according to our results, mixed economies are not a marginal choice, as they constitute 25% of the cases in our data sample. In addition, fishing seems to be a key element in the configuration of mixed economies, as it is present across all of them.

## Introduction

### The Origins of Agriculture

The Origins of Agriculture (OA) is a mainstay of archaeological research, being the transition to farming regarded as one of the major developments in our past [1–5]. Notwithstanding, after more than a hundred years of research on the OA, we are only just beginning to understand the details of the process [6], remaining plenty of questions unanswered and being the topic still considered one the most relevant scientific challenges for Archaeology [7].

Even though the adoption of agriculture has often been described as a rapid, unidirectional and inexorable process, the vast expansion of knowledge on the OA of the last two decades has shown that it is a complex phenomenon that encompasses a continuum of plant and animal management strategies; such a continuum frequently extended over long periods of time and involved an intricate interplay of environmental, social and cultural factors [8]. Therefore, the OA is no longer considered as a single monolithic research question, being now recognised as a higher order research domain comprising a wide range of different research questions, datasets, scales of analysis and analytical and theoretical approaches [9].

The most outstanding questions within the OA research field include its chronology and geography, as well as its causes, pace of development and spread, being all aspects deeply intertwined. Despite the existence—at a high level of detail—of almost as varied approaches to these questions as researchers writing about them, in more general terms, the OA state of play can be summarised around two main explanatory frameworks: traditional universalist explanations and more recent alternatives.

#### Traditional universalist explanations

As the adoption of agriculture occurred almost simultaneously in many different regions of the world, it has traditionally been explained in universalist terms, i.e., a single universal cause is proposed as the lever of the OA. The most renowned universalist prime-mover explanations may be summarised around three main paradigms:

1. **The superiority of agriculture as a mode of production**, irrespective of circumstances. Agriculture—a highly desirable development whose advantages were self-evident—would have been automatically adopted in favourable ecological conditions once the necessary knowledge—the limiting factor—had been reached [10]. Within this framework, growing agriculturalist populations would have colonised new territories, absorbing or displacing local hunter-gatherer groups, and making them adopt agriculture rapidly [11–15]. This, in turn, would have laid the ground for a series of socioeconomic changes that, later in time, resulted in urbanization processes and the emergence of civilisations [16–18].
  Accordingly, this first approach encompasses four main correlates that have long influenced the most widespread conception of the OA: (1) a conceptual dichotomy between “two mutually incompatible ways of life” [19]: hunting and gathering on the one side, agriculture on the other, with no intervening options [20]. Intermediate positions between the two are regarded as transitory, short-lived intermediate stages from one steady state to the other; over the long term, the great majority of populations are assumed to tend either to maintain a hunter-gatherer subsistence economy or to embrace agriculture, presenting a U-shaped distribution to the relative proportions of gathered/hunted versus produced food in the diet [21,22]. (2) The notion that the agricultural transition was a radical and rapid switch in human evolution, arising from an express domestication once the necessary knowledge was acquired [20,23,24], what enabled its consideration as a “revolution”: the Neolithic Revolution [25–27]. (3) The conception of the adoption of agriculture as a “point of no return” [17]; for those hunter-gatherer societies becoming agriculturalists, there would be no turning back unless they are compelled by environmental downturn or increased mortality [22]. And (4) a view of human history totally biased towards a classical idea of progress in which agriculture is clearly prioritised against hunting and gathering [18,25,28], hence being hunter-gatherers stereotyped as inherently simple and agriculturalists as culturally complex [29,30].
2. **Standard-Evolutionary-Theory-based (SET-based) explanations for initial domestication**. This second paradigm is based on the core assumption of unidirectional adaptation (environments change and organisms adapt, never vice versa [31]); more precisely, a population-resources imbalance is proposed as a direct or underlying cause of the transition to farming. Such an imbalance could have originated either on the supply side, the demand side or both [32,33]. Thereupon, domestication, intensification and/or the exploitation of suboptimal resources would have emerged as an adaptive response to the resource pressure induced by demographic changes in the form of a local [34,35] or a global [36,37] population increase, and/or by environmental changes such as a decline in resource availability [38–41] and/or climatic variations like the Pleistocene-Holocene climatic transition—which resulted in warmer, wetter conditions, a significant reduction in climate fluctuations, and a 33% increase in atmospheric CO_2_ [42–49].
  It is important to recall that the key assumption of this second paradigm is that hunter-gatherers would only have become agriculturalists under pressure, as farming is usually more labour-intensive, backbreaking and time-consuming than hunting and gathering [32], often leading to no immediate change in quality of life [32,50–52].
3. **Social hypothesis.** Under this paradigm, the onset of agriculture would have been motivated by social forces in stress-free scenarios. More precisely, a set of ‘social disequilibrium models’ [30,53–56] proposes that farming would have been embraced to maintain social control, or in the struggle for power, spouses and/or status [19]; this, in turn, would have resulted in the emergence of inequality and hierarchical societies.

#### More recent alternatives derived from EE, NCT and integrative proposals

Although evolutionary frameworks have been used to assess human behaviour for decades (see [57] as an example), their popularity has increased substantially in recent decades. More specifically, the latest explanatory approaches to the OA are either derived from Evolutionary Ecology (EE)—inclusive of Human Behavioural Ecology (HBE)—[4,58–60], from Niche Construction Theory (NCT) [2,3,9,61,62], or from integrative approaches comprising EE, NCT and models of cultural transmission and gene-culture coevolution that envisage all the perspectives involved as complementary, synergetic and broadening each other [58,63–69].

EE is a selectionist, neo-Darwinian high-level theoretical framework that provides a well-defined set of general concepts, assumptions and analytical tools—such as optimisation theory—for the study of specific adaptations through the lens of the interaction between evolutionary forces and ecological variables. One of its subfields, HBE, which investigates human behaviour in relation to ecological conditions and assesses the different behavioural strategies in terms of fitness [4,70], has already made substantial contributions to OA research [71]. In this regard, particularly famous is the Diet Breadth Model (DBM), according to which human groups are supposed to have a list of all the resources in their environment ranked in descending order of net caloric return, being delayed-return strategies—such as resource management and production—only embraced when immediate-return alternatives are not productive enough [4,69,72–75]. According to the DBM, at the dawn of agriculture, a resource depression would have forced the inclusion in the diet of previously ignored resources such as the wild ancestors of present domesticates (small to medium-sized mammals, seeds and tubers), resources otherwise falling beneath the “optimal diet” boundary of human societies world-wide [63].

On its part, NCT postulates that organisms are capable of modifying their own evolutionary trajectories—and those of other species—by actively engineering their living environments, which can provide them with evolutionary advantages [76]. Thence, according to NCT, domestication arose from large-scale human efforts at ecosystem enhancement in the absence of any sort of population-resources disequilibrium [2]. More specifically, NCT representatives [9,61,62,77,78] consider that agriculture would have emerged in climatically stable resource-rich scenarios (usually near water), where small-scale societies would have established small semi-permanent to permanent central settlements, and within which a wide range of plant and animal species would have been comprehensively auditioned over many generations, evolving just a subset of them into domesticates [2].

Over the last few years, the increasing wealth of data on the OA has evidenced that the transition to farming was characterized by a great variability across time and space, i.e., that the different world regions followed independent developmental pathways, being each one of them shaped by a number of complex and locally contingent factors, and generally extending over long periods of time [63,79]. As a result, considerable controversy has emerged regarding the utility of the different explanatory approaches for the OA, existing to this day no consensus on which approach is most appropriate; in fact, the OA community continues to be characterised by a lack of consensus in many respects [6], with just a few major areas of agreement that may be summarised as follows:

- At least eleven world regions have been identified as independent centres of domestication, but several more have been suggested [8]. In addition, each major independent region would have also included multiple loci for domestication [6,80].
- The unforeseen synchronicity in the timing of the first domesticates around the end of the Pleistocene [6,81]; more precisely, the earliest morphologically domestic cereals date to about 12,000–11,000 cal years B.P. [8]. However, it is not straightforward to identify when cultivation started, as new evidence is pushing the existence of pre-domestic cultivation—gathering of wild cereals and small-grained grasses—back to the late Pleistocene [46,82,83].
- The emergence of agriculture took place in resource-rich areas (instead of marginal ones as previously thought), which enabled domestication experiments and the sustained auditioning of different plant and animal species [77,81]. In fact, the cradles of agriculture are now regarded as the regions to which the most numerous and profitable future domesticates were native [84].
- The need to distinguish between OA, domestication and agriculture [6,8]. Albeit it is extremely difficult to define clear thresholds that separate the different stages, there are two confirmed delay phenomena that cannot be disregarded: (i) the existence of long periods of species manipulation/cultivation before the emergence of domestication traits (fixing the non-shattering spikelet in wheat, barley and rice took for instance up to ~2,000–4,000 years [85]); and (ii) the millennial-scale delay between initial domestication attested through morphological evidence and the development of fully agricultural economies [86,87]. Hence, domestication is not an automatic outcome of manipulation, and neither is agriculture of domestication, existing a vast middle-ground of long-term subsistence economies in between.
- The abandonment of traditional dichotomies between wild and domesticated, hunting-gathering and agriculture, etc., in favour of a continuum of plant and animal management strategies [6,63]. Such a continuum would stretch from hunting and gathering on the one margin, to fully agricultural economies on the other, encompassing all the above-mentioned middle-ground possibilities, and making no assumptions of progress or unidirectionality between them.
- Consistent with the above, the development of agriculture would have been a drawn-out process rather than a revolution [4,40,88–90].

In addition to the foregoing, a significant number of OA researchers agree that single causal scenarios based on prime movers fall short [3,8,63,79,91]. Clearly, the amelioration of climates at the end of the Pleistocene acted as a trigger; however, other relevant factors such as human demography, social systems and the biological characteristics of the auditioned species were operating simultaneously in tightly interconnected networks, thus being not possible nor convenient to select just one of them as the only cause. As a consequence, current approaches revolve around broader explanatory frameworks that try to integrate both the interplay of the different factors [3,6,8] and distinct theoretical approaches such as HBE and NCT [58,65,67–69].

For a succinct summary of the main theories on the OA described above and the most relevant references, please refer to the S1 Table the Supplementary Material.

### The vast middle-ground

In the previous section, we concluded by underscoring the inherent complexity of the transition to farming, which stems mainly from the protracted nature of the process, its marked variability across time and space, the multitude of factors involved at both the macro and micro scales, the intricate nature of the diverse middle ground, and the subsequent long-standing debates on the suitability of the different explanatory approaches. As pointed by Smith [86], from all the factors mentioned, the middle ground is probably the most largely neglected one, a fact that continues to hamper significantly our current understanding of the agricultural transition. Hence, in the present paper we intend to shed light on what the middle ground may have looked like. Given that the interest of the present contribution may be best understood in the light of what is known about the middle ground thus far, here we proceed to cover some of the key aspects.

The middle ground refers to the conceptual territory between hunting, gathering and fishing on the one side, and agriculture and husbandry on the other. In accordance with the classic dualistic epistemology, foragers had no domesticates, and all human societies with domesticates had agriculture, being the transition between the two regarded as a mere shift [86]. Consistently, the middle ground has traditionally been regarded as some sort of hodgepodge of all the subsistence economies not meeting the criteria to be considered either hunter-gatherers or agriculturalists. Nevertheless, as discussed earlier, there is a global consensus that such dichotomic approaches are both insufficient and inefficient for several reasons, outstanding among which are (i) the difficulty of defining “hunter-gatherers” and “agriculturalists”: over the years, numerous definitions have been proffered (some of them inspired in real referents coming from Ethnography and/or Archaeology, whose attributes have been established as diagnostic); however, hitherto no single definition is considered to have a global or universal applicability [92,93]. (ii) The prolonged nature of the transition to agriculture, which indeed extended over millennia, hence having had to encompass many successful long-term subsistence alternatives that are fully disregarded in the binary approach [86]. And (iii) its implicit strait-ahead linear sense, which depicts the transition as an evolutionary process irreversibly leading to agriculture [94]. Therefrom, while the conceptual dichotomy can still be useful as chronological markers that delimit the extremes of the middle ground, it is also quite harmful, since it blurs and oversimplifies the multiple changes in subsistence behaviour that must have occurred during the Holocene in many different regions of the world. Moreover, even if different adjectives such as “complex” hunter-gatherers, “affluent” foragers, “incipient” agriculturalists, etc., have been used in an attempt to differentiate middle-ground strategies, those expressions still hold the imprint of the binary conceptualisation, and implicitly displace in-between societies to one margin or the other, hence concealing again the relevance of the middle ground in its own right.

More recently, different authors have addressed the middle ground in various ways that transcend the dichotomous perspective. In overall terms, their proposals can be grouped around two main approaches: compartmental schemes and non-classificatory perspectives. As stated by Smith [86], compartmental schemes are aimed at “identifying and defining categories of human-plant and human-animal interaction” characteristic of a given subset of middle-ground societies. When taken together, all these interaction categories “form a continuum of increasing human intervention or involvement in the life cycle of targeted species […] that encompasses the landscape that lies between hunting-gathering and agriculture”.

Over the years, many scholars have adopted the compartmental approach: see for instance, Ford [95], Harris [96–99], Higgs [100] and Zvelebil [24,101] (among others); however, the most renowned of all compartmentalists is decidedly Bruce D. Smith thanks to his proposal in [86] that if the binary categories food procurement and food production are maintained, the latter should be split into three main sub-categories: *low-level food production without domesticates*, *low-level food production with domesticates* and *agriculture*. Smith encompasses under food production all human actions aimed at intervening in the life cycle of targeted species, regardless of the presence or absence of domestication traits. Within the food production territory, subsistence strategies reliant on domesticates for less than a 30%–50% of the annual caloric intake are considered low-level food production, while those above such boundary are deemed fully agricultural economies. Regarding the partition between low-level food production without domesticates and low-level food production with domesticates, as their name suggests, the boundary marker is domestication, i.e., the presence of morphological/genetic changes associated with the domestication syndrome. Nonetheless, as recurrently highlighted by Smith [86], the definition of boundaries is an extremely troublesome task, since manifold management strategies have been documented across all categories at different intensity levels. Therefore, the relevance of Smith’s proposal is not to be found in the robustness of the definition of boundaries, but on the creation of the low-level food production category and its two subcategories; a conceptualisation that serves to highlight the importance of such a diverse array of stable subsistence strategies, whose persistence attests to their versatility and success in a wide range of different socioenvironmental contexts.

Several ensuing publications incorporated Smith’s conceptualisation of the middle-ground either making use of the term *low-level food production* [3,87,102,103] or developing similar ones such as: *low-level food-resource producers* [104,105] or *mixed economies* [4,106–110]. Notwithstanding, other authors such as Terrell et al. [93] have argued that instead of a classification system whose inter-category boundaries are difficult to establish, it would be more appropriate to describe and compare subsistence strategies without first having to label them. Hence, they proposed the “provisions spreadsheet”, an interactive matrix of species and harvesting tactics intended to overcome restrictive definitions of domestication, and to accommodate the known diversity of human subsistence practices. Notably, the provisions spreadsheet covers four different aspects: goal (food or raw materials), primary variables (resource breadth and their yield, accessibility and reliability), secondary variables (behavioural or environmental manipulation skills necessary to exploit those resources) and observations, to faithfully reflect the particularities of each socio-economic strategy and of the environment within which they develop(ed).

To this day, no-one of the two approaches has proven to be better than the other. However, this is not necessarily a problem, as both proposals serve to place on the middle ground the emphasis it deserves, and highlight the difficulty of establishing clear-cut frontiers between the different subsistence possibilities. In fact, in the last decades, plenty of empirical evidence—both ethnographic and archaeological—is showing that a great deal more research is needed to understand the different subsistence combinations, and to unravel the particulars of the middle ground. Regarding the most outstanding recent findings on the middle ground, particular mention must be made of the innate complexity of hunter-gatherer resource management techniques, the sophistication of complex hunter-gatherer societies and the relevance of mixed economic choices.

As regards hunter-gatherer complexity, several recent contributions have served to emphasise both the diversity inherent in hunter-gatherer subsistence practices, and the great variety of their socio-political systems [111–113]. One of the major advances has certainly been the recognition that no human societies are *simple* by definition, as even the smallest hunter-gatherer societies present intricate social relationships and elaborated economic solutions that comprise scheduling, mobility, task differentiation, gender division of labour, etc. [112]. In addition, the engagement in efforts at landscape engineering such as regular burning, tending, tilling, transplanting, weeding, sowing and selective harvesting has been documented for both past and present hunter-gatherers and low-level food producers, which supports the existence of a wide spectrum of hunter-gatherer and low-level-food-production subsistence strategies without clear frontiers between them [109,114,115]. Particularly outstanding among the different landscape modification techniques is the so-called slash-and-burn cultivation, a common practice in tropical forested areas that consists in cutting the trees and undergrowth of the selected site, leaving them to dry for a few months, and eventually burning them to restore minerals and nutrients to the soil prior to sowing—which is done at the beginning of the rainy season [116]. In the Amazon basin, for instance, slash-and-burn is widely reported in the ethnographic record in connection with manioc and/or maize cultivation; in addition, numerous works point to its use in pre-Columbian times as either an adaptation of late Holocene immigrating agriculturalists or as an extension of Amazonian house gardens. Be that as it may, the fact is that Amazonia hosts Holocene age archaeological evidence for anthropogenic landscape modification via burning, the vast majority of which is located in the vicinity of natural concentrations of aquatic resources, which underscores the well-known Amazonian complementarity between fish and cultivated plants [117].

From all the above, hunter-gatherers are no longer regarded as failed agriculturalists living in marginal environments; quite the opposite, at present, hunting-gathering and low-level food production are conceptualised as a broad range of successful long-term socioeconomic solutions characterised by their flexibility, resilience and adaptability to very different contexts [113].

As regards mixed economies, plenty of past and present empirical examples attest to their great variety and endpoint solution status. In the context of Archaeology and Prehistory, mixed economies tend to refer to economies developed during the Mesolithic and the Early and Middle Neolithic. Although some publications refer to mixed economies whenever fishing complements hunting and gathering [118], most researchers designate under mixed economies a combination of foraging (hunting, gathering and/or fishing) with different food-production activities such as farming at different intensity levels and/or small-scale herding [119–122]; more precisely, the most widespread definition of a mixed economy is probably the one proffered by Winterhalder and Kennet [60] as “an economy (…) that includes, in long-enduring combinations, both foraging and the low-level use of cultivars or domesticates“.

Some of the most paradigmatic archaeological examples of mixed economies include the Jomon (Japan) and Chulmun (Korea), whose resource procurement strategies included hunting, gathering, fishing and resource production strategies ranging from annual plant encouragement and tree management, to domestication of particular species and cultivation of others [105,123–125]; the Okhotsk (preceded in time by the Jomon), who hunted, gathered, raised pigs and had a few crops [105]; the peoples from the Northwest Coast of North America (typically classified as complex hunter-gatherers), who were strongly reliant on mariculture and conducted plenty of resource management activities to sustain and enhance both marine and plant resources: beach clearance, clam and root gardening, transplanting salmon eggs, tree modification, landscape burning, soil tilling, etc. [126–129]; and the late Prehispanic peoples from Sierras of Córdoba (Argentina), whose subsistence economy was a mix of small-scale farming and broad-scale foraging [120].

In the ethnographic record, some of the most well-known examples of mixed economies include the Ituri forest foragers of the Democratic Republic of Congo (who engage in partnership relationships with agriculturalists), the Agta from the Philippines (famous for their exchange of meat for rice), the savanna Pumé (a Venezuelan mobile hunter-gatherer society that incorporates manioc cultivation as a fallback strategy) [106], the Mlabri (skilled hunter-gatherers now living in north-eastern Thailand and the western Laos, who establish symbiotic trading relationships with more settled groups) [130], and present-day indigenous communities in the Arctic (whose economy combines foraging with trade or other economic activities such as full-time or part-time paid work, seasonal labour, craft-making, commercial fishing and/or tourism) [107,130–132].

It is clear from the foregoing that the study of the middle ground is key to understand prehistoric societies in general, and the transition to agriculture in particular. Remarkably, both in the ethnographic and archaeological records, we count with plenty of examples that illustrate the whole economic variability that human societies have implemented around the globe for millennia. In this regard, it is important to recall that even if Ethnography and Archaeology tend to deal with different time periods and rhythms [133], and being fully aware that available ethnographic information does not necessarily and directly inform about prehistoric societies, the fact is that ethnographic data allows to test archaeological theory by confronting it with real case studies. Concretely, as regards subsistence strategies, ethnographic examples enable to identify which economic choices are plausible, and in which contexts they are effectively taking place, thus contributing to a general theory of human and social behaviour that can be used as a frame of reference for prehistoric studies [134,135].

### Research proposal

From a methodological perspective, our contribution is framed within the increasingly relevant research line of integration, mining, analysis and interpretation of archaeological, ethnographic and anthropological data to explore global patterns through advanced computer-based approaches [136–138].

In particular, in the present work we intend to explore the middle ground by means of a comprehensive analysis of the subsistence strategies of 1290 societies extracted from the Ethnographic Atlas [139–141], which are either historical or ethnographically documented. The purpose of such an approach is twofold: (i) increasing our understanding of the different combinations of subsistence strategies that have been developed across the world over time; and (ii) providing new perspectives from which to explore prehistoric economies, the persistence of hunter-gatherer(-fisher) economies, and the agricultural transition. Note that we are not aiming at establishing direct formal analogies between our data and Prehistory, but at better understanding human economic choices, the contexts in which they develop, and their success in the long term. Therefore, our study can be considered to belong to the theory-building realm, and it may help to look at prehistoric economies in a new light, and/or to hypothesise in a different way about particular archaeological contexts.

More precisely, we are interested in the following research questions:

1. **Regarding subsistence strategies, are all combinations viable or do specific patterns exist?** The rationale behind this question is to be found in the fact that human societies configure their economic choices so as to procure enough food for group survival and a balance of required nutrients; such a balance is usually attained by including in the diet different foodstuffs coming from both the plant and animal realms. However, the selection of those foodstuffs is not a trivial undertaking, as the choice is influenced by a series of variables including—among others—resource availability (their temporal and spatial distribution), population density, the degree of technological development, etc. In other words, human subsistence choices both depend on and modify the carrying capacity of the whole socio-ecological system, being therefore relevant to explore if all combinations are feasible or if only a subset of them are successful. In this line, it will also be of interest to look into the possible complementary or exclusive relationships that could exist between the different subsistence strategies.
2. **The role played by ecological settings in the configuration of the different subsistence economies**, as they may prevent specific economic choices while fostering others. For instance, in some settings the carrying capacity can be increased through greater labour investment and/or diversification without the need to adopt new strategies, while in others intensification is unfeasible because of environmental reasons (e.g. agriculture is impracticable in Arctic areas and deserts). Therefore, (and without falling into environmental determinism), one may expect the association of specific economic choices with certain ecosystems (or biomass richness), hence being of interest to look for those relationships in empirical data.
3. **The role of fishing in the development of viable alternatives to cultivation.** Coastal and riverine areas constitute bountiful ecosystems with a specific behaviour regarding carrying capacity and resource availability. In these contexts, the only limitations are a priori technological and/or related to biogeographical constraints, since resources are abundant and resource restoration is in principle not problematic. Henceforth, in such settings, the development of *ad hoc* technologies expedited the emergence of successful long-term subsistence economies that, in some cases resulted in sedentism, large population densities, intensive exploitation of resources and increased social complexity, traits long assumed to be exclusive of agricultural societies [114]. Thus, the study of economies in which fishing has a significant weight may be useful to understand non-agricultural successful economic choices and/or to shed light on the middle ground.

## Materials and methods

### Data sources

All data used in the present study were extracted from the Database of Places, Language, Culture, and Environment—D-PLACE—[138] accessible at https://d-place.org/. In words of its creators, D-PLACE is “an attempt to bring together the dispersed corpus of information describing human cultural diversity” [142], being its main goal to facilitate cross-cultural analyses. More specifically, D-PLACE integrates information from multiple datasets and presents it in a unified and consistent manner.

The sample of societies selected is that of the Ethnographic Atlas (EA) [139–141], which in its original form includes up to 1291 pre-industrial societies [143] ranging from small hunter-gatherer groups to societies with complex agricultural economies—i.e., market economies are not included. However, in the present contribution we reduced the EA sample size to 1290, since that is the number of societies for which we had information on subsistence strategies. It is important to note that while these societies are globally distributed, the EA has especially good coverage of Africa and western North America [144], and that as a consequence of the pre-industrial nature of the societies in the EA, the conclusions obtained from the analyses in this paper cannot be extrapolated to market economies. As regards the focal time period, the different time frames and the percentages of EA societies falling under each of them can be found in Table 1.

**Table 1:** Focal time periods of the societies documented in the EA.

Focal time period	% of EA societies
Before 1800	3
1800–1899	27
1900–1950	66
After 1950	2

From Table 1 it becomes clear that the time span covered is quite circumscribed. This aspect is particularly convenient, since it avoids problems associated with longer time frames, namely changing ecological and social conditions, different occupations of the same site over time, long-term transitions in subsistence economies, social organisation, etc.

The different subsistence-related and socio-ecological variables used in this work and the datasets from which they were extracted are detailed in Table 2. Note that the column Data type indicates if the variable is originally continuous, categorical or ordinal in D-PLACE. Subsequent transformations of the variables are duly explained in the corresponding Rmarkdowns available at the GitHub repository of the paper: https://github.com/Virahe/Lets-go-Fishing.

**Table 2:** Variables selected for the present study from the different sources available at D-PLACE.

Dataset	Variables			
	D-PLACE variable ID	Short name	Description	Data type
Ethnographic Atlas (Ethnology, 1962; Gray, 1998; Murdock, 1967)	EA001	% dependence on gathering	Percentage of dependence on the gathering of wild plants and small land fauna, relative to other subsistence activities.	Ordinal
	EA002	% dependence on hunting	Percentage of dependence on hunting, including trapping and fowling, relative to other subsistence activities.	Ordinal
	EA003	% dependence on fishing	Percentage of dependence on fishing, including shellfishing and the pursuit of large aquatic animals, relative to other subsistence activities.	Ordinal
	EA004	% dependence on husbandry	Percentage of dependence on animal husbandry, relative to other subsistence activities.	Ordinal
	EA005	% dependence on agriculture	Percentage of dependence on agriculture, relative to other subsistence activities.	Ordinal
	EA028	Agriculture intensity	Intensity of cultivation. Levels: no agriculture, casual, extensive/shifting, horticulture, intensive, intensive irrigated.	Categorical
	EA029	Major crop type	Principal type of crop cultivated. Levels: no agriculture, non-food, vegetables, tree-fruits, roots/tubers, cereals.	Categorical
	EA030	Settlement patterns	The prevailing type of settlement pattern. Levels: nomadic, seminomadic, semisedentary, impermanent, dispersed homesteads, hamlets, villages/towns, complex permanent.	Categorical
	EA033	Jurisdictional hierarchy beyond local community and political complexity	The number of jurisdictional levels beyond the local community, with 1 representing the theoretical minimum (none/autonomous band or villages) and 4 representing the theoretical maximum (villages nested within parishes, districts, provinces, and a complex state). This variable also provides a measure of political complexity, ranging from 1 for stateless societies, through 2 or 3 for petty and larger paramount chiefdoms or their equivalent, to 4 or 5 for large states. Imposed colonial regimes are excluded.	Ordinal
	EA039	Plow cultivation	Indicates whether or not animals are employed in plow cultivation, and whether plow cultivation is aboriginal or dates to the post-contact period. Levels: absent, not aboriginal but present, present.	Categorical
	EA040	Type of domestic animals	The predominant type of animals kept. Levels: absence or near absence, pigs, sheep/goats, equine, deer, camelids, bovine.	Categorical
	EA041	Milking	Indicates whether or not domestic animals milked. Levels: absence or near absence, more than sporadically.	Categorical
	EA202	Population size	Population of ethnic group as a whole. Note that source differs by society; EA bibliography is source where possible, otherwise Ember [145].	Continuous
Jenkins et al. [146]	AmphibianRichness	Amphibian richness	Number of coexisting amphibian species.	Continuous
	BirdRichness	Bird richness	Number of coexisting bird species.	Continuous
	MammalRichness	Mammal richness	Number of coexisting mammal species.	Continuous
Kreft and Jetz [147]	VascularPlantsRichness	Vascular plant richness	Number of coexisting vascular plant species.	Continuous
Moderate Resolution Imaging Spectroradiometer [148]	AnnualNetPrimaryProductionVariance	Variance in net primary production per month	Variance in net primary production per month.	Continuous
	MonthlyMeanNetPrimaryProduction	Net primary production per month (grams of carbon uptake per square meter of land per month)	Net primary production per month (grams of carbon uptake per square meter and land per month).	Continuous
	NetPrimaryProductionConstancy	Net primary production constancy	Colwell’s [149] information theoretic index. Indicates the extent to which a climate patterns are predictable because conditions are constant. Varies between 0 (completely unpredictable) and 1 (fully predictable).	Continuous
	NetPrimaryProductionContingency	Net primary production contingency	Colwell’s [149] information theoretic index. Indicates the extent to which a climate patterns are predictable because conditions oscillate in a very predictable manner. Varies between 0 (completely unpredictable) and 1 (fully predictable).	Continuous
	NetPrimaryProductionPredictability	Net primery production predictability	Colwell’s [149] information theoretic index. Indicates the extent to which a climate patterns are predictable due to either constancy or contingency. Varies between 0 (completely unpredictable) and 1 (fully predictable).	Continuous
Terrestrial Ecoregions of the World	Biome	Biome	Classification by Dinerstein et al [150] of the earth into fourteen units that host similar formations of plants and animals due to their climates. A single biome can be found over a range of continents.	Categorical
Baseline Historical (1900–1949), CCSM ecoClimate model [151]	AnnualMeanTemperature	Mean value of monthly temperature across the year	Mean value of monthly temperature across the year	Continuous
	AnnualPrecipitationVariance	Variance in monthly precipitation means	Variance in montly precipitation means	Continuous
	AnnualTemperatureVariance	Variance in monthly temperature means	Variance in monthly temperature means	Continuous
	MonthlyMeanPrecipitation	Mean monthly precipitation in ml/m^2^/month	Mean monthly precipitation in ml/m^2^/month	Continuous
	PrecipitationConstancy	Precipitation constancy	Colwell’s [149] information theoretic index. Indicates the extent to which a climate patterns are predictable because conditions are constant. Varies between 0 (completely unpredictable) and 1 (fully predictable).	Continuous
	PrecipitationContingency	Precipitation contingency	Colwell’s [149] information theoretic index. Indicates the extent to which a climate patterns are predictable because conditions oscillate in a very predictable manner. Varies between 0 (completely unpredictable) and 1 (fully predictable).	Continuous
	PrecipitationPredictability	Precipitation predictability	Colwell’s [149] information theoretic index. Indicates the extent to which a climate patterns are predictable due to either constancy or contingency. Varies between 0 (completely unpredictable) and 1 (fully predictable).	Continuous
	TemperatureConstancy	Temperature constancy	Colwell’s [149] information theoretic index. Indicates the extent to which a climate patterns are predictable because conditions are constant. Varies between 0 (completely unpredictable) and 1 (fully predictable).	Continuous
	TemperatureContingency	Temperature contingency	Colwell’s [149] information theoretic index. Indicates the extent to which a climate patterns are predictable because conditions oscillate in a very predictable manner. Varies between 0 (completely unpredictable) and 1 (fully predictable).	Continuous
	TemperaturePredictability	Temperature predictability	Colwell’s [149] information theoretic index. Indicates the extent to which a climate patterns are predictable due to either constancy or contingency. Varies between 0 (completely unpredictable) and 1 (fully predictable).	Continuous

### Methods: Data analysis

The analysis of the database described in the previous section was performed in two stages:

1. Exploratory data analysis by means of unsupervised learning techniques: Principal Components Analysis and clustering.
2. Supervised learning approach.

The details of the analytical methods applied in each of the two stages are summarised below.

### Exploratory data analysis by means of unsupervised learning techniques

Unsupervised learning is a subfield of machine learning conceived to look for patterns in a dataset with no predefined response variable, i.e., it is referred to as unsupervised since no output variable guides the analyses [152,153]. Under the umbrella of unsupervised learning we find a great variety of methodologies, many of which seek to find relationships either between variables or between observations, hence being generally applied within the framework of exploratory data analysis. Regarding the present contribution, we have drawn on two of the most commonly used unsupervised learning techniques, namely Principal Components Analysis (PCA) and clustering, to address our first research question: *Are all subsistence combinations viable or do specific patterns exist*?

#### Principal Components Analysis (PCA)

The principal components of a dataset are the dimensions in feature space along which the original data present the greatest variation; these dimensions also define the lines and subspaces that are closest to the data cloud (minimum squared distance). More precisely, for a dataset *X* with *n* observations and *p* features (all standardised to have mean zero and standard deviation one), each component is a linear combination of the *p* features, and all components together constitute an orthonormal basis, which ensures that they are linearly uncorrelated [152].

PCA is the unsupervised learning technique consisting in the computation of the principal components and their subsequent use in understanding the data. It is frequently used for dimensionality reduction and visualisation purposes, since by keeping just the first few principal components and projecting all data points onto them, a low-dimensional representation that captures as much of the information as possible is obtained. Note that the interpretation of the different components is generally conducted by projecting the features on the new low-dimensional space, and by thereafter assessing the correlations between the different features and the corresponding components.

In the context of the present study, we took all the societies in the EA, focused exclusively on the variables ranging from EA001 to EA005, (i.e., the percentages of dependence on gathering, hunting, fishing, husbandry and agriculture, what we have called the subsistence dataset), and performed PCA to ascertain whether the variability in our data could be explained by just a few components, and if so, to identify which of the variables had a more significant role in explaining such variability.

#### Clustering

The term clustering encompasses a broad set of algorithms aimed at identifying groups (clusters) in a dataset, so that the observations within each group share some common features, while observations in different groups are quite different from each other [152,154]. In relation to our first research question, should only a subset of subsistence economies be successful and persistent, it would translate into the data having cluster structure. Therefore, at this stage of the analyses, we took again the subsistence dataset and conducted cluster analysis to ascertain whether it had structure, and if so, to explore the subgroups identified among the observations.

More specifically, we first assessed the clustering tendency of the subsistence dataset following Kassambara [155]; then, we opted for the hierarchical clustering methodology [152], since instead of committing to a specific number of clusters, it results in a dendrogram that allows conducting analysis at different levels of resolution; and finally, we tried to determine the optimal number of clusters—the height at which to cut the dendrogram—by means of the multiple techniques proposed in [155] and consensus clustering [156].

#### Assessment of clustering tendency

A noteworthy issue in clustering analysis is that clustering techniques will return clusters even if the data do not contain any meaningful clusters. Therefore, before applying any clustering algorithm, it is necessary to check whether the data contain non-random structures. To that end, Kassambara [155] proposes two alternatives:

- **Statistical methods: Hopkins statistic (*H*).** The Hopkins statistic estimates the probability that a given dataset has been drawn from a random uniform distribution (null hypothesis).
- **Visual methods.** The Visual Assessment of cluster tendency (VAT) algorithm [157] computes the dissimilarity matrix between the observations in the dataset, and rearranges it so that similar objects are close to one another; its output is the ordered dissimilarity image (ODI). Typically, one computes the ODI of both the real dataset and a random dataset generated from it, so that the results can be compared.

#### Hierarchical clustering

As its name would suggest, hierarchical clustering is a clustering technique intended to build a hierarchy of clusters of the observations in a dataset [152]. Its main advantages are (i) that it results in a dendrogram (an intuitive tree-based representation of the observations); and (ii) that it is not necessary to specify the number of clusters in advance, as the dendrogram covers all possibilities from the lowest level—at which each cluster contains a single observation—to the highest one—at which all the observations belong to the same cluster. Consequently, hierarchical clustering is extremely useful to conduct analysis at different levels of resolution, which is precisely the reason why we chose it to explore the different subsistence economies. Nevertheless, it is noteworthy that not committing to a number of clusters beforehand can also be regarded as a drawback, namely the lack of an objective criterion to determine the number of clusters, which can result in the biases of the analyst influencing the choice.

#### Determination of the optimal number of clusters

Once the data have proven to be clusterable and the clustering algorithm has been selected, the next step is trying to ascertain the optimal number of clusters. However, there is no one answer to this question, as the optimal number of clusters depends on both the partitioning algorithm chosen and the similarity/dissimilarity metric used. In fact, there exists a great variety of indices and methods conceived to determine the optimal number of clusters, see, for instance, the elbow method [155], which selects the number of clusters that minimises the total intra-cluster variation; the average silhouette method [158], which maximizes the quality of clustering, i.e., how well each observation lies within its cluster; the gap statistic [159], which chooses the clustering furthest away from the random uniform distribution of points; all the indices included in the NbClust R package [160]; and consensus clustering approaches [156]. For further details on the NbClust R package, please refer to its reference manual available at [161]. As regards consensus clustering, it is a resampling-based method designed to determine the consensus number of clusters in the data, and to assess and represent cluster stability. In particular, consensus clustering takes the original dataset, creates a given number of perturbed datasets via the resampling technique of choice, applies the clustering algorithm selected to those perturbed datasets, and finally assesses the agreement among the multiple runs. Its underlying assumption is that the more robust clusters are to sampling variability, the more likely it is that they reflect the real structure of the data.

In the present work, we opted for computing all the above-mentioned metrics and selecting the best number of clusters either in accordance with the majority rule, with consensus clustering and/or with both.

#### Cluster entropy

After choosing the number of clusters, in the present contribution we have drawn on the concept of information entropy to formally define mixed economies. Within the framework of information theory, the entropy of a random variable is defined as the uncertainty intrinsic in the variable’s possible outcomes [162]. Formally, the entropy of a discrete random variable *X* is calculated as follows:

$$H\left(X\right)=-\sum_{i=1}^{n}P\left(x_{i}\right){log}_{b}P\left(x_{i}\right)$$ (1)

Where *X* = {*x*_1_,⋯,*x*_*n*_}, i.e., *x*_1_,⋯,*x*_*n*_ are the possible outcomes of *X*, and they occur with probability *P* (*x*_1_),⋯,*P* (*x*_*n*_). Note that the entropy is maximal when all possible outcomes are equiprobable (uniform probability distribution).

In the context of the present contribution, a subsistence economy is defined by five different variables—namely the percentages of dependence on gathering, hunting, fishing, husbandry and agriculture—that are interpreted as the probability of relying on those food sources. Therefore, given that the maximal entropy is attained when all possible outcomes follow a uniform distribution, the subsistence economies with the same percentage of dependence on the five alternatives will be the ones with maximal entropy. Consequently, mixed economies can be formally defined as those subsistence combinations with high/the highest information entropy.

### Supervised learning approach

Supervised learning techniques are intended for the type of problems in which each observation—codified as a vector with the corresponding values for the different predictor variables—has an associated response measurement. In particular, supervised learning methods seek to fit a model that relates the response to the predictors, either for prediction purposes or to better understand the relationships between the response and the predictors [152].

Supervised learning problems can be, in turn, divided into two main categories based on their response variable: regression problems (quantitative response) and classification problems (qualitative response); specific machine learning techniques have been developed for each problem typology, existing also methods that can be used for both quantitative and qualitative responses.

As regards the present contribution, once structure has been found in the data, and the prevailing subsistence economies have been identified via clustering (i.e., the number of clusters has been selected and hence each observation has been assigned to a cluster), we can use such information as the response variable to conduct supervised learning analyses. More precisely, we have opted for supervised learning approaches to address our second research question, i.e., *to assess the role played by ecological settings in the configuration of the different subsistence economies*.

First, it is necessary to clarify that this second stage of the analyses has been conducted on what we called the supervised learning database: a dataset including all the variables in Table 2 except for the percentages of dependence on the different subsistence strategies (variables from EA001 to EA005), plus the response variable (the cluster to which each observation belongs); note that we have chosen the response variable for *k* = 15, which is the highest level of resolution. Therefore, given that our response variable is qualitative, we are facing a classification problem. Notably, we did not include variables from EA001 to EA005 in the supervised learning database because the clustering analysis has been conducted exclusively on the five of them, and thus, they are closely related to the response variable, which could hinder the identification of other possible relationships between the rest of socioecological variables and the clusters found.

When joining the variables in Table 2 to configure the supervised learning database, we found that we had missing data, i.e., that certain societies in the EA had no information available for some of the variables coming from other data sources. To solve this problem, instead of dropping those societies with missing values, we kept all of them and applied a multiple imputation technique (as opposed to single imputation) to estimate the statistical uncertainty attributable to the missing data. This approach consists in creating multiple complete datasets by independently imputing the missing data via stochastic draws from the distributions of the observed data. In particular, in our work we have used the Multiple Imputation by Chained Equations (MICE) method [163], which is implemented in the *mice* R package [164]. MICE assumes that data are missing at random (MAR)—i.e., that the probability that a value is missing depends only on the observed data—and consists in iteratively estimating the conditional distributions of each variable from the rest of the variables. To increase stability, the imputation process of all variables is repeated through cycles. In our analysis, we have generated 100 different datasets, being each one of them the result of 50 cycles. As for the MICE imputation method chosen, we have used a recursive partitioning approach: random forests [165,166], so as to take into account possible nonlinearities, interactions and both numerical and categorical data. Recall that even though variables from EA001 to EA005 have not been considered for the supervised learning analyses, we did include them in the database on which multiple imputation was conducted, so as to help get more accurate estimates of the missing values.

Regarding the supervised learning model to be fitted to our data, from all techniques suited for classification problems, we have chosen random forest [167]; the rationale behind this choice is to be found in four main reasons: (i) as previously stated, random forest can handle both numerical and categorical predictors; (ii) it is an ensemble method, which translates into the resulting model having a good bias-variance trade-off—i.e., the resulting model is neither too general nor overfitting the data [152,153]; (iii) in an exhaustive evaluation of up to 179 classifiers, random forest was found to be the best family of classifiers [168]; and (iv) it allows to conduct both individual and group variable importance analyses. Nevertheless, to be sure that our choice was appropriate, we have compared random forest with several benchmark and highly performant classification algorithms. More specifically, we have conducted an ANOVA test and the Duncan’s New Multiple Range Test [169] on the accuracies obtained by stratified 10-fold cross-validation with the following classifiers: ZeroR [170], OneR [171], AdaBoost [172], Support Vector Machine with polynomial kernel [152,173], rotation forest [174] and random forest [167]. Note that in the Duncan’s New Multiple Range Test, if two classifiers belong to the same group, no statistically significant differences exist between them, which implies that the choice of the classifier can be based on criteria other than accuracy—see for instance interpretability, model simplicity, etc.

As far as variable importance analyses are concerned, their raison d’être is that predictors are seldom equally important in supervised learning models; in fact, usually only a subset of them is relevant to determine the response. Therefore, it is of interest to quantify the relative contribution of each variable (individual variable importance analysis) or group of variables (group variable importance analysis) in predicting the response. In particular, variable importance is calculated as the mean decrease in accuracy after randomly permuting a given predictor [167] or a group of predictors [175]. Recall that the more the accuracy is reduced after the permutation, the more important the predictor/group of predictors.

Remarkably, variable importance analyses become even more important in the context of ensemble models, since ensembles result into better accuracy than single-tree models, but they do so at the expense of interpretability.

## Results

### Exploratory data analysis by means of unsupervised learning techniques

#### PCA

The results of the PCA conducted on the subsistence dataset (variables from EA001 to EA005) can be found in Table 3, where we can see that the total variance of the data is effectively explained by the first four PCA dimensions.

**Table 3:** Results from PCA on the subsistence dataset.

PC Dim.	1	2	3	4	5
Eigenvalues	2.624	1.043	0.842	0.492	0.000
% of var.	52.477	20.851	16.841	9.831	0.000
Cumulative % of var.	52.477	73.328	90.169	100.000	100.000

The first four dimensions explain all the variance in the data.

**Fig 1:**
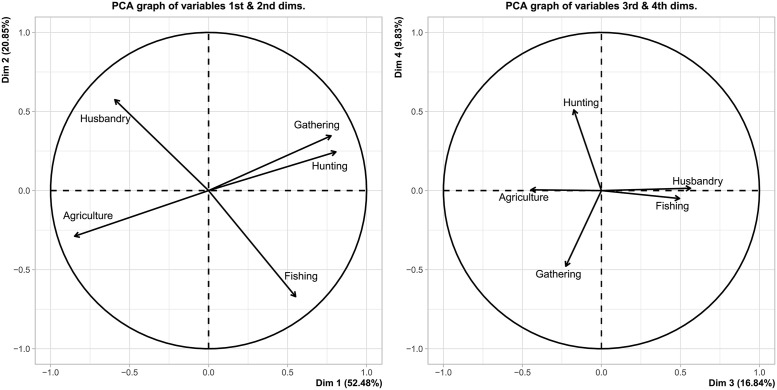
PCA graph of variables for dimensions 1:2 and 3:4 respectively.

In Fig 1 we can see how the different variables considered (percentage of dependence on gathering, hunting, fishing, husbandry and agriculture) correlate with the first four PCA dimensions. The actual correlation values and their respective *p*-values can be found in S2 Table from the Supplementary Material.

A contextualised interpretation of these four PCA dimensions may be summarised as follows:

- Dimension 1 explains 52,48% of the variance. In overall terms, it corresponds to the traditional opposition between hunting-gathering and agriculture. The immediate interpretation of such result is that at least for our EA sample, the traditional dichotomic view of subsistence economies explains only half of the picture.
- Dimension 2 explains 20,85% of the variance. Once each society has positioned itself to one side or the other of the previous hunting-gathering vs. agriculture divide, dimension 2 presents an additional disjunctive, in this case between animal husbandry and fishing. Therefore, the interpretation thus far would be that—in general terms—agriculturalists do not hunt or gather, and that if you fish, you do not practise animal husbandry (and vice versa). In addition, it is important to note that in accordance with the relative position of husbandry and fishing in the PCA graph of variables for dimensions 1:2, husbandry generally accompanies agriculture, while fishing is most frequently found complementing hunting-gathering economies.
- Dimension 3 explains 16,84% of the variance. This third dimension presents an opposition between agriculture and husbandry & fishing. A plausible reading of its meaning would be that once a society is profiled in accordance with the two previous disjunctives, dimension 3 informs about the intensification choice, i.e., if they intensify agriculture (plant resources) or husbandry/fishing (animal resources).
- Dimension 4 explains 9,83% of the variance. The divide it presents is between gathering and hunting, and hence it may be understood as follows: for those societies who positioned themselves as hunter-gatherers in the previous dimensions, dimension 4 captures if they intensify gathering or hunting.

### Assessment of clustering tendency

**Fig 2:**
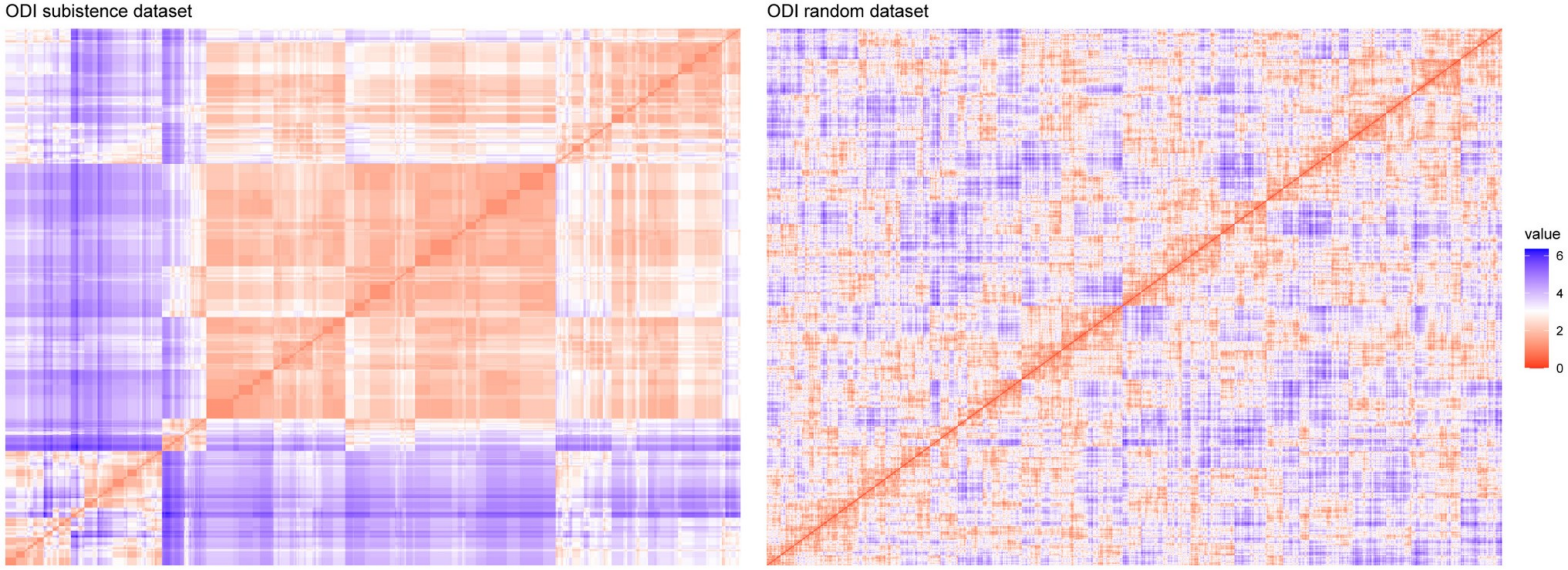
ODIs of the subsistence dataset (on the left) and a random dataset generated from it (on the right). Note that according to the legend colour red means high similarity (low dissimilarity) and blue high dissimilarity. After comparison with the random dataset, it becomes clear that our subsistence data have structure.

- **Statistical methods: Hopkins statistic (*H*).** As previously stated, Hopkins statistic tests the spatial randomness of the data. An *H* value close to 0.5 is in perfect agreement with the null hypothesis that the dataset was generated by a uniform distribution. On the other hand, an *H* value close to zero means that the data are highly clusterable, i.e., the null hypothesis can be rejected. Our subsistence dataset has an *H* value of 0.1527 (significantly below the threshold of 0.5), which means that our data are highly clusterable.
- **Visual methods. The Visual Assessment of cluster tendency (VAT) algorithm.** In Fig 2. ODIs of the subsistence dataset (on the left) and a random dataset generated from it (on the right). Note that according to the legend colour red means high similarity (low dissimilarity) and blue high dissimilarity. After comparison with the random dataset, it becomes clear that our subsistence data have structure. we can see the ODIs obtained for both the subsistence dataset and a random dataset generated from it, using the Euclidean distance as dissimilarity metric. From the comparison of the two, it becomes clear that our subsistence dataset presents a clear structure and that it is therefore significantly different from the random one.

#### Hierarchical clustering

Having shown in the previous section that the data are clusterable, here we proceed to explain the hierarchical clustering implementation that we applied to our subsistence dataset. We have worked with the R package *factoextra* [176], and more precisely, we have used the the *eclust* function selecting the Euclidean distance as dissimilarity metric, and Ward’s method as the agglomerative method.

#### Determination of the optimal number of clusters

As emphasised in the Methods section, one of the main strengths of hierarchical clustering—and the reason why we chose it—is that it enables to conduct analyses at different scales of resolution. Accordingly, in the present contribution we have decided to explore the resulting dendrogram at three levels: *k* = 2, 7 and 15. The reasons justifying these choices may be summarised as follows:

- *k* = 2 was chosen since it is the optimal number of clusters proposed by NbClust following the majority rule, and it is also the optimal value according to the elbow method and the average silhouette method; additionally, the consensus matrix for *k* = 2 happens to be extremely clean, which is an indicator of good clustering. For the details of the values obtained for the different indices please refer to S3 and S4 Tables in the Supplementary Material. As regards the consensus matrices obtained with consensus clustering, for a visual inspection of them please refer to the folder *Consensus matrices & CDF* available at the GitHub repository of our paper: https://github.com/Virahe/Lets-go-Fishing. Note that in such visual representation, each consensus matrix has been arranged so that items belonging to the same cluster are adjacent to each other; consequently, perfect consensus corresponds to a diagonal matrix with non-overlapping blocks along the diagonal.
- *k* = 7 and *k* = 15 were chosen after the joint analysis of the consensus matrices, the evolution of the cumulative distribution functions (CDF’s) as *k* increases, and the increase in the area under the CDF for the different *k*’s. Recall that bimodality in the CDF is considered as a signal of cluster structure, and that if *k* < *k*_*TRUE*_ the area under the CDF increases with *k*, but once *k*_*TRUE*_ is reached, any further increase in *k* does not translate into a significant increase in the area under the CDF. Taking into account all the above, we assessed the evolution of the CDF’s and of the delta function (both showed in S1 Fig from the S1 Appendix in the Supplementary Material), and found that there are two distinct levels worth exploring: (i) the one ranging from *k* = 5 to *k* = 10, and (ii) that ranging from *k* = 11 up to *k* = 20. Both levels have been analysed at the intermediate value of the intervals, i.e., *k* = 7 and *k* = 15 respectively. For further details on the choice of *k* = 7 and *k* = 15 please refer to S1 Appendix in the Supplementary Material.

#### Detail of the clusters obtained for *k* = 2, 7 and 15

In the present section we succinctly interpret the clusters obtained at the three levels selected. 3. **Sunburst of the different subsistence strategies identified via hierarchical clustering for *k* = 2, 7, 15. A short description is provided for each of the clusters. Note that the size of the circular sectors is proportional to the number of societies falling under each cluster.** is a sunburst diagram that summarises the cluster precedence relationships. Note that the lowest level of detail (*k* = 2) is to be found in the inner circle, and that the greatest detail (*k* = 15) is presented in the outermost circle. Recall as well that the percentage values accompanying the cluster descriptions are the mean values obtained for the given variable across all societies in that cluster.

At the lowest level of resolution, we find the traditional dichotomy between hunting-gathering-fishing and agropastoralism. When moving to the intermediate level, the differences between the distinct subsistence alternatives become more evident: the hunter-gatherer-fisher (HGF) group is divided into three depending on which of the three activities is more heavily relied upon: (1) gathering, (2) hunting or (3) fishing; as for the agropastoralist group, it is splitted into four clusters: (4) the strictly pastoralists, whose strategy is based predominantly on animal husbandry and complement it with a low percentage of agriculture; (5) the agropastoralists, who are strongly dependent on agriculture and complement their subsistence economy with animal husbandry; (6) the group which we have agreed to name *agrofishers* since their subsistence economy is a mix of fishing and agriculture, being the rest of possible activities—hunting, gathering and husbandry—underrepresented; and (7) those with what we have called the *Whole Spectra Economy* (WSE), i.e., whose subsistence economy encompasses hunting, gathering and fishing at approximately par value, and a great percentage of agriculture.

Eventually, at the highest level of resolution we identify the more subtle nuances according to which the previous seven clusters are further subdivided. As for the three HGF clusters, they are partitioned into six clusters for *k* = 15: the HGF’s more dependent on gathering are divided into two clusters: (1.A.) those who are just hunter-gatherers (HG’s) more reliant on gathering (we claim that they are simply HG’s since fishing represents a negligible value in this cluster); and (1.B.) the archetypal HGF’s with a similar percentage of dependence on each of the three sources; the group of HGF’s more heavily reliant on hunting is maintained and now named (2.BIS.); as regards the cluster of HGF’s more heavily reliant on fishing, they are splitted into three clusters: (3.A.) the eminently fishers, with a percentage of dependence on fishing greater than 70%; (3.B.) hunter-fisher groups with similar percentages of dependence on each of the two strategies and a negligible level of gathering; and (3.C.) HGF’s–Fishers, who in spite of being more heavily dependent on fishing resources, present significant percentages (greater than 20%) of gathering and hunting. Concerning the other four clusters (the ones with a given dependence on agriculture), for *k* = 15 they have been further splitted as follows: the pastoralist cluster has been divided into two different groups: (4.A.) the outstandingly pastoralists, which in this smaller cluster present average values of up to a 75% dependence on animal husbandry and just a 13% complementation with agriculture; and (4.B.) agropastoralists that are more strongly dependent on animal husbandry than in any other source (up to 49% on average), and that complement their strategy not only with agriculture (22%) but also with hunting (12%) and fishing (11%), what allows to consider them as having a mixed economy. Regarding the agropastoralist cluster, it has been divided into three clusters: (5.A.) the strictly agropastoralists—again—whose percentages of dependence on both agriculture and husbandry are almost maintained with respect to its homonymous cluster at *k* = 7; (5.B.) a second group of agropastoralists more heavily reliant on animal husbandry; and (5.C.) what we have called *agrohunting*, which is still heavily reliant on agriculture, but instead of complementing the subsistence economy with just husbandry, it also presents a significant degree of hunting (in fact, the percentage of dependence on hunting is greater than that of husbandry). As for the *agrofishing* cluster, it has been partitioned into two clusters in accordance with the percentages of dependence on agriculture, fishing and husbandry: (6.A.) *agrofishers* with an average 47% reliance on agriculture, almost 40% dependence on fishing and a marginal contribution of husbandry to the diet of up to 7%; and (6.B.) *agrofishers* more strongly dependent on agriculture (54%), with a 20% percentage of dependence on fishing, and a greater weight of husbandry (13%). Lastly, the cluster of the WSE has been splitted into two: (7.A.) the truly *agrohunters*, which exhibit an average 45% reliance on agriculture, 28% on hunting and complement their strategy with an 18% of fishing; and (7.B.) the strictly WSE, which is not significantly altered with respect to its homonymous cluster at *k* = 7, presenting now an average reliance on agriculture of up to 35% and of 21% on hunting, 25% on gathering and 15% on fishing.

At this point, it would be interesting to recall that even though for *k* = 2 there is no trace of mixed economies, and at *k* = 7 they are restricted to two clusters (agrofishers and the WSE), when we increase the level of detail and work at a fine-grained scale (*k* = 15), they make up a significant percentage of the possible subsistence economies, in fact, 5 out of 15 clusters can be considered as mixed: clusters (4.B.), (6.A.), (6.B.), (7.A.) and (7.B.). For further details on this aspect, please refer to the discussion, where it is examined in depth.

**Fig 3:**
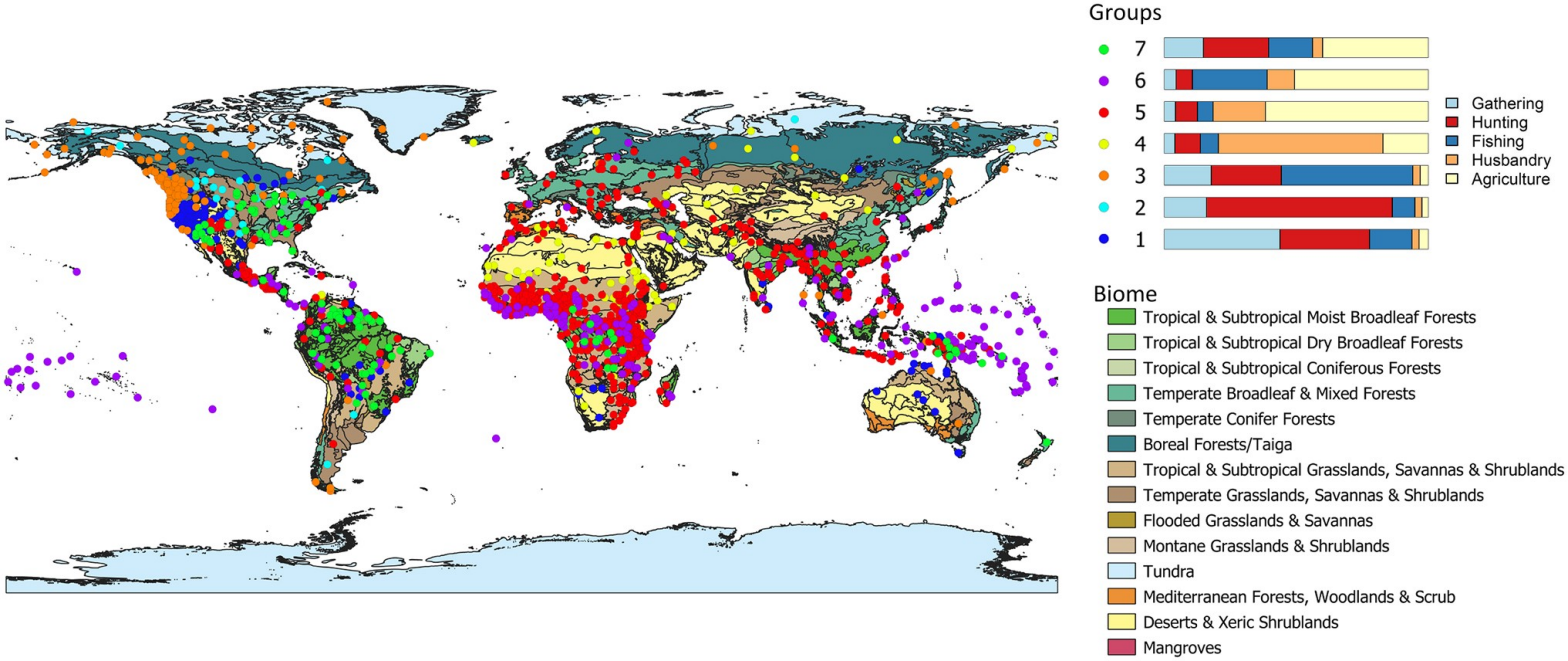
Map with the subsistence clusters obtained for *k* = 7. The different societies have been placed in accordance with their latitude and longitude and coloured according to the cluster they belong to. The different world regions have been coloured to represent the different biomes. Two legends provided: the upper right one presents the detail of the average percentages of dependence on gathering, hunting, fishing, husbandry and agriculture of each of the clusters. The lower right one is the legend of the biomes. Map source–[150]. Biome cartography is licensed under CC-BY 4.0 (https://ecoregions2017.appspot.com/).

**Fig 4:**
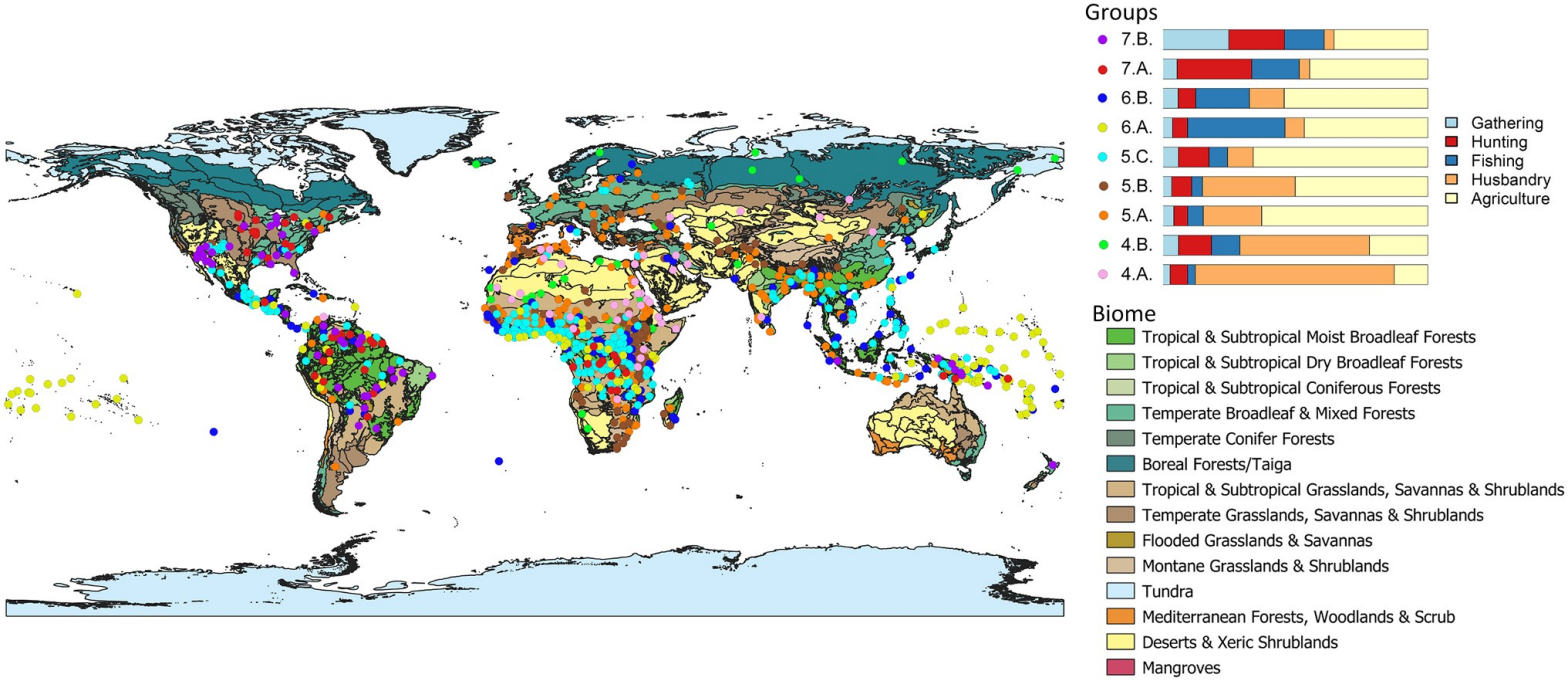
Map with 9 of the subsistence clusters obtained for *k* = 15. The different societies are placed in accordance with their latitude and longitude and coloured according to the cluster they belong to. Only those subsistence strategies with a significant level of agriculture and/or husbandry have been considered. Two legends provided: The upper right one presents the detail of the average percentages of dependence on gathering, hunting, fishing, husbandry and agriculture of each of the clusters. The lower right one is the legend of the biomes. Map source–[150]. Biome cartography is licensed under CC-BY 4.0 (https://ecoregions2017.appspot.com/).

Additionally, we decided that it could be of interest to represent the different clusters in a map with the terrestrial ecoregions of the world proposed by Dinerstein et al. [150]. Therefore, we created Figs 3 and 4. In Fig 3 we can see all societies considered in the present study placed in accordance with their latitude and longitude and coloured according to the cluster they belong to for *k* = 7; note that the map has intentionally been coloured to represent the different biomes, and that a legend with the average percentages of the different variables for each cluster has been provided. As for Fig 4, it is the replication of Fig 3 for *k* = 15, with the caveat that instead of representing the 15 clusters, as the interest of the present paper is on the agricultural transition and the middle ground, we have just represented the 9 clusters with a given level of dependence on agriculture and/or husbandry.

#### Cluster entropy

As stated in the Methods section, in this work we have formally defined mixed economies as those subsistence strategies with high/the highest information entropy—remember that maximal entropy is achieved when all possible outcomes follow a uniform distribution, which in our case would translate into subsistence economies with the same percentage of reliance on the five variables considered (gathering, hunting, fishing, husbandry and agriculture). Note that in our results, the WSE is the subsistence economy closest to the case with maximum theoretical entropy.

**Fig 5:**
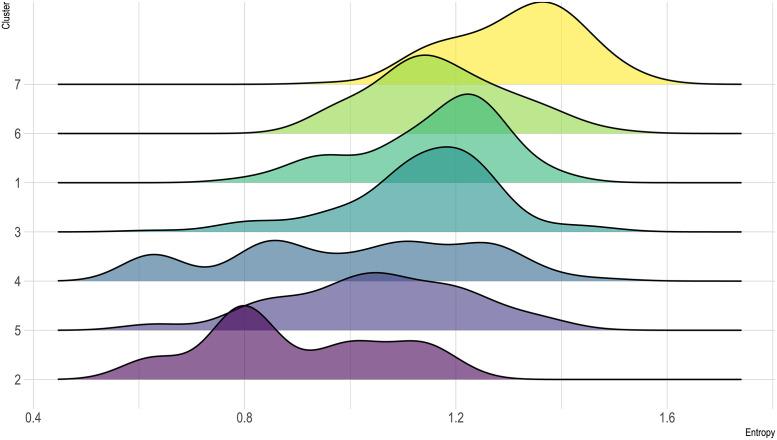
Ridgeline plot of the entropy distributions of each cluster for *k* = 7. Recall that the distributions have been sorted in ascending order of entropy along the vertical axis.

To illustrate the concept, we have calculated both the entropy of each society’s subsistence strategy and the entropy of each cluster’s average strategy for *k* = 2, 7 and 15. (Recall that a cluster’s average strategy is calculated by averaging the percentages of dependence on gathering, hunting, fishing, husbandry and agriculture across all societies in the cluster). For each level of analysis, we have obtained: (i) a summary table with the clusters’ average strategies, their entropy, their standard deviation, two columns quantifying the number of variables with a percentage of dependence equal or greater than 15% and 10% respectively, and a concise interpretation of each cluster; as well as (ii) a figure with the entropy distributions of all the clusters in that resolution level. For simplicity, here we just present the results obtained for *k* = 7, which are provided in Table 4 and Fig 5 respectively. Nevertheless, the detail of the entropy results obtained for the other two levels of resolution can be found in the Supplementary Material S5 Table and S1 Fig. for *k* = 2, and S6 Table and S2 Fig for *k* = 15.

**Table 4:** Summary table for *k* = 7.

	Clusters’ average strategies (Mean values per variable and cluster)									
Cluster nb	Gathering (%)	Hunting (%)	Fishing (%)	Husbandry (%)	Agriculture (%)	Entropy	SD	Limit 15	Limit 10	Interpretation
2	16,40	69,82	8,50	2,64	2,64	0,95	28,42	2	2	HGF–Hunters
5	4,74	8,21	5,91	19,67	61,47	1,14	23,93	2	2	Agriculture(62%) + Husb.(20%)
4	4,43	9,51	6,77	61,89	17,40	1,15	23,92	2	2	Husbandry(62%) + Agric.(17%)
3	18,16	26,36	49,42	2,73	3,33	1,22	19,27	3	3	HGF–Fishers
1	44,00	33,64	16,00	2,64	3,73	1,24	18,33	3	3	HGF–Gatherers
6	4,77	6,16	28,20	10,30	50,57	1,25	19,49	2	3	AgroFishing
7	15,21	24,55	16,46	3,80	39,99	1,42	13,40	4	4	WSE: HGF + Agriculture

It includes cluster number, each cluster’s average strategy (the average of the percentages of dependence on gathering, hunting, fishing, husbandry and agriculture across all societies in the cluster), their entropy, standard deviation, the number of variables with a percentage of dependence equal or greater than 15% and 10%, and a succinct interpretation of the cluster. Note that the table has been sorted in ascending order of entropy.

### Supervised learning approach

#### Comparison of different benchmark and highly performant classification algorithms

As stated in the Methods section, we have compared random forest (our intended algorithm of choice) with several benchmark and high-performance classification algorithms on our supervised learning database; the scale of resolution selected has been *k* = 15, since it is at this level that the diversity of the middle ground strategies becomes more evident. Note that since the supervised learning database had missing data, and hence we obtained 100 imputed datasets via the MICE multiple imputation method, the comparison of the algorithms has been conducted on the 100 imputed datasets.

As we explained, the classifiers selected were the following: ZeroR, OneR, AdaBoost, Support Vector Machine (SVM) with polynomial kernel, rotation forest and random forest. ZeroR and OneR are rule-based classifiers typically used to establish the baseline performance, as ZeroR trivially predicts the most frequent class, while OneR creates one rule for each of the predictors in the dataset and establishes as its ‘one rule’ the one with the smallest classification error. The rest of the classifiers chosen are high-performant classifiers [168,177]. Our experiments have been conducted in Weka [170]; stratified 10-fold nested cross-validation was used for parameter tuning in AdaBoost and the SVM with polynomial kernel (for the details of the parametrizations explored please refer to our GitHub repository: https://github.com/Virahe/Lets-go-Fishing); as regards rotation forest and random forest, in accordance to [177] tuning makes no significant improvement to them as long as reasonable parameter values are set; therefore, given that parameter tuning is extremely time-consuming, we used the parametrization they propose in Table 2, and evaluated the two algorithms by means of simple stratified 10-fold cross-validation.

To assess if statistically significant differences existed between the six classifiers on our data, we conducted one-way ANOVA tests on the 10-fold cross-validation accuracies obtained for the six of them on each imputed dataset. In all the 100 cases the null hypothesis of equality of means could be rejected. Thereupon, having proved that statistically significant differences existed between the algorithms, we conducted the Duncan’s New Multiple Range Test—again on the results for the 100 datasets—to get the detail of the differences. In all the 100 cases, random forest belonged to group “a”, the group with the highest performance. In 99 out of 100 datasets group “a” was composed by random forest, rotation forest and the SVM with polynomial kernel. In the remaining dataset, random forest belonged to group “a”, rotation forest to group “ab”, and the SVM with polynomial kernel to group “b” ─recall that classifiers with the same letter are not statistically significantly different. Hence, we can conclude that since random forest belongs to the group with the highest performance in 100 out of 100 of the datasets, it is perfectly valid to justify its choice on grounds of the variable importance analyses it enables to conduct.

#### Variable importance analyses

As we have already discussed, since random forest is not straightforwardly interpretable, it is of interest to conduct variable importance analyses to assess the relevance of a given variable or a group of variables in predicting the response. In coherence with the previous section, variable importance analyses have been conducted for *k* = 15.

#### Individual variable importance analysis

**Fig 6:**
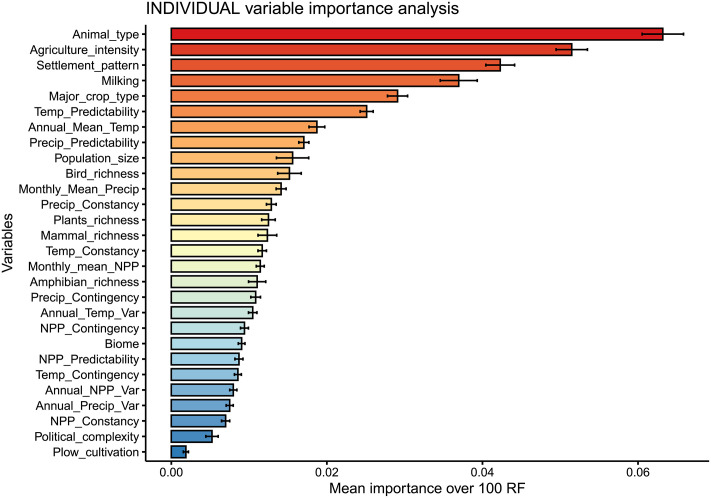
Breiman’s individual variable importance averaged across the 100 imputed datasets. One standard deviation error bar.

We have conducted individual variable importance analysis on the 100 imputed datasets. The results provided in Fig 6 show the importance of each variable averaged across the 100 datasets. Remember that we have conducted individual variable importance analysis in accordance to Breiman [167], and that therefore, the importance of each variable is the percent increase in misclassification rate obtained after randomly permuting the values for that variable.

#### Group variable importance analysis

Group variable importance analysis is intended to assess the impact of an aggregated set of variables in the resulting predictive accuracy of the random forest algorithm. In the present contribution, four groups of variables have been considered. Please find below the detail of the variables that constitute each group.

*Group 1 –Agriculture-related variables*:
  - EA028 –Agriculture intensity.
  - EA029 –Major crop type.
  - EA039 –Plow cultivation.
*Group 2 –Husbandry-related variables*:
  - EA040 –Domestic animals’ type.
  - EA041 –Milking.
*Group 3 –Demographic/degree of complexity variables*:
  - EA030 –Prevailing type of settlement pattern.
  - EA033 –Political complexity.
  - EA202 –Population size.
*Group 4 –Ecological variables*:
  - Variables from Jenkins et al. [146]:
    - Amphibian richness.
    - Bird richness.
    - Mammal richness.
  - Variables from Kreft and Jetz [147]:
    - Vascular plants richness.
  - Variables from Moderate Resolution Imaging Spectroradiometer [148]:
    - Annual Net Primary Production Variance.
    - Monthly Mean Net Primary Production.
    - Net Primary Production Constancy.
    - Net Primary Production Contingency.
    - Net Primary Production Predictability.
  - Variables from Terrestrial Ecoregions of the World [178]:
    - Biome.
  - Variables from Baseline Historical (1900–1949), CCSM ecoClimate model [151]:
    - Annual Mean Temperature.
    - Annual Precipitation Variance.
    - Annual Temperature Variance.
    - Monthly Mean Precipitation
    - Precipitation Constancy.
    - Precipitation Contingency.
    - Precipitation Predictability.
    - Temperature Constancy.
    - Temperature Contingency.
    - Temperature Predictability.

**Fig 7:**
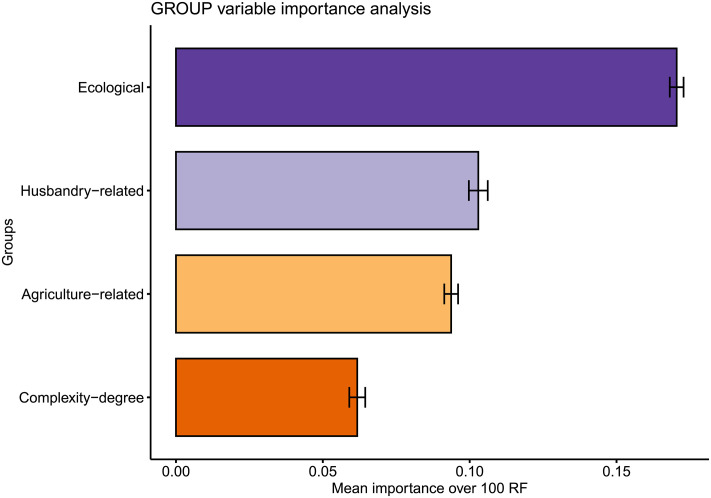
Group variable importance averaged across the 100 imputed datasets. One standard deviation error bar.

The results of our group variable importance analyses averaged across the 100 imputed datasets can be found in Fig 7. Group variable importance averaged across the 100 imputed datasets. One standard deviation error bar.

## Discussion

In spite that standardised databases for the Digital Humanities in general, and all those available at D-PLACE [138] in particular, are not exhaustive, they are representative enough of human societies’ variability and allow to identify previously uncharted regions, hence being particularly suitable for cross-cultural studies such as the present one. Nevertheless, it is also noteworthy that since the contents in these databases are commonly quite generalist, one should be extremely careful when extrapolating conclusions to specific cases.

That said, we proceed to revise our three research questions in the light of the results obtained.

### Question 1: Regarding subsistence strategies, are all combinations viable or do specific patterns exist?

This question has been specifically addressed by means of the unsupervised learning approach; the answer we found is that clear patterns exist in the data, hence not being all combinations viable. More specifically, the PCA analysis has allowed us to identify the four main axes of variability in the subsistence database—which explain up to 100% of the variance—and the data have proved to be highly clusterable.

As regards PCA analysis, remarkably, the first PCA dimension—which explains up to 52.48% of the variability—correlates quite clearly with the traditional dichotomy between hunting-gathering and agropastoralism. Thereupon, we can assert that at least for our particular EA sample of 1290 societies, the binary conceptualisation has proved to be both useful and insufficient. It is indeed useful as it explains approximately half of the variance in our data, but it is at the same time insufficient as it leaves an equally important half unexplored. Assuming that our sample may be in fact representative enough of the entire phenomenon, this finding could suggest that because of embracing the traditional dichotomic approach, half of the picture could have been disregarded.

Additional PCA-derived highlights would be—as noted in the Results section—(i) the exclusive relationship found between fishing and animal husbandry in dimension 2, which corresponds to the fact that societies with a high reliance on fishing generally do not have the need to breed livestock and vice versa (i.e., pastoralists are mostly found in arid areas with no access to fishing resources); and (ii) the disjunctive from dimension 3 between agriculture and husbandry-fishing, which is clearly related to intensification strategies, namely, if it is decided to intensify on plant resources or on animal protein.

Regarding cluster analysis, the fact that the subsistence dataset is significantly different from a random distribution and highly clusterable implies that recurrent specific subsistence combinations exist. As stated in the methods section, we opted for hierarchical clustering because it allows to explore the problem under consideration at different scales. More precisely, the three levels of granularity selected (*k* = 2, 7, 15) could be regarded as corresponding to the macro-scale, the mesoscale and a more detailed scale respectively.

The macro-scale—which provides a general overview of the phenomenon—finds again the traditional division between HGF and agropastoralists; this fact complements and reinforces the reading that we made in the context of the percentage of variance explained by the first PCA dimension: that the classic binary conceptualisation corresponds to a broad-brush approach, hence being quite oversimplistic and incomplete. For this reason, here we will focus primarily on the mesoscale, since it is detailed enough to distinguish the different subsistence strategies, while being at the same time general enough so as to get a global view of the matter. Nevertheless, when of interest, we will also delve into the specifics of certain strategies at the greatest level of detail, i.e., for *k* = 15.

#### Cluster interpretation for *k* = 7

Recall that in the present section, in addition to exploring the economic structure of each of the clusters found, we will also provide some insights in relation to the biomes where they appear. For the details on geographical location and entropy values, unless otherwise specified, please refer to Fig 3 and Table 4 respectively.

### HGF strategies

- **Cluster 1 (136/1290 societies): HGF–Gatherers–H(34%)G(44%)F(16%)**
  This cluster corresponds to a gathering-hunting economy in which wild plant management constitutes the most relevant activity. Societies belonging to cluster 1 are predominantly located in areas with deserts and xeric shrublands biome (Australia, South Africa, NW coast of the North American continent), existing also some cases in the biomes tropical and subtropical grasslands, savannas and shrublands, and boreal forests.
  The prevalence of gathering in arid areas—which may seem counterintuitive—has its rationale in the fact that plants collect water, being hence an extremely valuable resource for water obtention in those environments. Examples of the implementation of these techniques can be found in the literature for Australia—where aboriginal communities extract water from the trunk of the cajuput-tree [179]—and the Kalahari desert—where melons constitute an important source of moisture [180]. The existence of such arid-areas-management dynamics has been considered in Archaeology—and is still being explored—as an explanation for the human habitation of desertic environments [181,182].
- **Cluster 2 (24/1290 societies): HGF–Hunters–H(70%)G(16%)F(9%)**
  Cluster 2 corresponds to a hunting economy that is marginally complemented with plant gathering and fishing. It tends to appear in northern Subarctic areas—Siberia, Alaska, Nunavut territory in Canada—where the predominant biome is tundra, as well as in the temperate grasslands, savannas and shrublands of central North America. As far as Arctic areas are concerned, in these ecosystems seasonality reduces plant production during part of the year, which explains why middle and big game constitute the primary food source for their inhabitants. In addition, secondary products of big game hunting, such as fat and oil, bones and skins, etc., are first need products given the low temperatures of those territories [183].
- **Cluster 3 (115/1290 societies): HGF–Fishers–H(26%)G(18%)F(49%)**
  Cluster 3 presents a foraging economy heavily reliant on fishing that is complemented with hunting; plants play a minor role in this group. The majority of societies from cluster 3 are geographically located in cold coastal areas whose biome is either tundra or taiga: northern Japan, southernmost South America (Tierra del Fuego), Arctic and Subarctic areas (Russia, northern Canada, Alaska), which explains why gathering is so marginal. Also remarkable is the agglomeration of societies from this cluster that we find in the NW coast of North America (with a temperate conifer forest biome). Notably, waters in all these areas are extremely rich; Northern Pacific coasts, for instance, are part of the highest primary producer waters in the world [184]. Such richness of fishery products together with the fact that winters in most of these territories translate into scarcity of terrestrial resources, justify the subsistence choice made by their peoples. Circumpolar and Inuit communities are paradigmatic examples of this cluster.

### Farming strategies

- **Cluster 4 (78/1290 societies): Pastoralists–Husbandry (62%) + Agriculture (17%)**
  Cluster 4 corresponds to the pastoralist strategy, i.e., that in which animal husbandry is the activity clearly prioritised. Such a great reliance on animal husbandry is combined with a 17% of dependence on agriculture (either to supplement human diet or to obtain fodder for their herds). The geographical location of the pastoralist societies in our EA sample points to an almost exclusive correlation between this subsistence economy and the deserts and xeric shrublands biome. Moreover, it would seem that pastoralism is an African-Asiatic strategy, since while it is the predominant subsistence economy in the arid lands from North and South Africa, the Arabian Peninsula, Kazakhstan and Mongolia, it has almost no presence in Europe, America and Oceania. Additionally, as it could be expected, most pastoralist societies are either nomadic or seminomadic.
- **Cluster 5 (613/1290 societies): Agriculturalists–Agriculture (62%) + Husbandry (20%)**
  Cluster 5 corresponds to the eminently agricultural strategy and it is by far the biggest one, encompassing almost 50% of the societies in our EA sample. Its distribution of percentages mimics that of the pastoralist cluster but in reverse, i.e., agriculture is the food source most heavily relied upon, and it is complemented with just a 20% of animal husbandry. Agriculturalist societies are present in the following biomes: tropical and subtropical moist broadleaf forests (Oceania, Southeast Asia, China, Bangladesh, India, Pakistan, the African Sahel, Central America, the external area of the Amazon basin); tropical and subtropical grasslands, savannas and shrublands as well as flooded grasslands and savannas (Central and South Africa); Mediterranean forests, woodlands and shrub (Mediterranean basin); temperate broadleaf and mixed forests (Central Europe and NE coast of North America); and temperate conifer forests (SW North America). Undoubtedly, agriculture is viable in a wide range of environments whose common denominator is that all of them are either tropical or warm areas with water availability.
  It is also worth highlighting that an overwhelming majority of the societies in the present cluster have cereals as their major crop type, i.e., cereals constitute their food staples. Notably, even though agriculture in general—and staple agriculture in particular—customarily correlate with demographic increase, being as well regarded as the necessary condition for the emergence of sedentarism and hierarchical societies, it is also a fact that staple agriculture substantially increases both labour needs and socioeconomic risk [185]; as a result of relying so heavily on a single economic activity, agricultural societies are known to experience more frequent famines than hunter-gatherers [186].

### Mixed strategies

- **Cluster 6 (226/1290 societies): *Agrofishing*–Agriculture (51%) + Fishing (28%) + Husbandry (10%)**
  Cluster 6 presents a mixed economy in which half of the food resources are obtained from agriculture, constituting fishing the second major food source; this subsistence strategy is further completed with a minor contribution of animal husbandry. The type of agriculture practised by most societies in this cluster is horticulture, being roots/tubers and tree-fruits the most frequent crop types.
  As it can be seen in Fig 3, the *agrofishing* strategy corresponds mainly to the biome of tropical and subtropical moist broadleaf forests. Remarkably, a relevant part of the societies is located in islands; more precisely, the islands in which we find *agrofishing* include Taiwan, the Philippines, Indonesia, most of the islands from Micronesia, Melanesia and Polynesia, as well as Madagascar and the Caribbean archipelagos. Additionally, we find *agrofishing* in Vietnam, Burma, Bangladesh, India, the African Sahel, Central America and the external area of the Amazon basin. Lastly, *agrofishing* communities are also found in the context of the African Great Lakes, where the biome is either flooded grasslands and savannas or tropical and subtropical grasslands, savannas and shrublands. The fact that this subsistence strategy is primarily found in coastal and riverine tropical areas may suggest that *agrofishing* is the result of the specific dynamics of those environments, namely their resource richness—recall that aquatic environments constitute some of the highest productivity areas in the planet [187]—and their high climatic stability and resource predictability [188]. Eventually, an additional remark would be the geographical overlap that exists in various regions between *agrofishing* and the agricultural strategy (see for instance the Philippines, Indonesia, Vietnam, India, the Sahel and the African Great Lakes area, Central America and the external area of the Amazon basin) which may denote the possibility of resource exchange between agriculturalists and *agrofishers*, a fact already suggested by several authors [130,189].
- **Cluster 7 (98/1290 societies): *Whole Spectra Economies* (WSE)–H(25%)G(15%)F(17%) + Agriculture (40%)**
  Cluster 7 corresponds to what we have called *Whole Spectra Economies* (WSE), i.e., a subsistence economy that encompasses the two ends of the continuum—foraging on the one side and agriculture on the other—both with a similar relevance. More specifically, societies with a WSE present a 40% reliance on agriculture, being the distribution of percentages between the three foraging strategies—hunting, gathering and fishing—quite even. As far as location is concerned, WSE appear in a wide range of different environmental contexts: (i) the tropical and subtropical moist broadleaf forest biome: Papua New Guinea, the African Sahel, Central America, the peripheral territories of the Amazon basin; (ii) the biome of flooded grasslands and savannas: African Great Lakes and the Parana basin; (iii) the deserts and xeric shrublands biome: Gulf of California; (iv) the biome of temperate broadleaf and mixed forests: New Zealand, the American Great Lakes region and the Mississippi basin; and (v) the temperate conifer forest biome: Louisiana and the Florida Peninsula. The very different contexts in which WSE are found attest to their flexibility and adaptability to diverse circumstances. In particular, WSE are probably the most resilient of all the strategies found, given that their main defining trait is diversification, which might translate into a significant reduction of the risk of food shortages and famines.

Thus far we have analysed the seven clusters found at the mesoscale, and our main conclusion is that they could be divided into two main groups: *primary economies* and *mixed economies*. More precisely, we have realised that it is the internal distribution of dependencies on the different subsistence strategies that holds the key to distinguishing between the classical binary categories and the middle ground. This is because the configuration of such internal distribution ultimately informs about the different types and degrees of specialisation. Even though specialisation is usually defined in relation to the exploitation of a narrow subset of resources [190], here we refer by specialisation to exhibiting high percentages of dependence—around 50%—on one to two subsistence strategies. Therefore, under *primary economies* we refer to those subsistence economies showing a strong reliance (i.e., a high specialisation) on one to two foraging or farming strategies—recall that for the case of two dominant strategies, both must be either foraging or productive. Consequently, the categories derived from the traditional HG-versus-farming divide fit reasonably well into this definition. Remarkably, in accordance with our results 75% of the societies in the EA present a primary economy, stemming the differences between the distinct primary-economy clusters from the strategy that is prioritised (if hunting is given priority over gathering/fishing, agriculture over animal husbandry, etc.). In this vein, it is also important to note that although the HG-agropastoralist disjunctive is already established by the first PCA dimension, further PCA dimensions have been considered for the identification and characterisation of the different clusters falling under primary economies.

On the other hand, we refer under *mixed economies* to all those subsistence economies that combine both foraging and productive strategies, and that generally rely on a wider spectrum of subsistence strategies (typically three or four). Additionally, as stated in the methods section, mixed economies can alternatively be defined as a function of the information entropy; more specifically, as a consequence of their reliance on a greater number of subsistence strategies, mixed economies correspond to those alternatives with higher/the highest information entropy. Unquestionably, the most distinguishing feature of mixed economies is the combination of foraging and farming within the same subsistence economy, something which does not occur among the primary economies, and which turns them into extremely valuable examples of what the middle ground may have looked like. Ultimately, it is remarkable that up to 25% of the societies in our EA database have a mixed economy; such a relevant percentage proves once again that instead of a single intermediate category between hunting-gathering and agriculture, mixed economies constitute a complex phenomenon worthy of study in its own right. Consequently, since one of the aims of the present contribution is to gain a better understanding of human subsistence variability in general, and of the middle ground in particular, hereunder we proceed to explore the particularities of the mixed economies in greater detail (for *k* = 15).

#### Mixed economies for *k* = 15

As we saw in the Results section, at *k* = 15 we find further divisions of the previous seven clusters that help profile more clearly the particularities of the different alternatives. However, as previously stated, here we will limit our discussion to those economies that we have defined as mixed.

**Fig 8:**
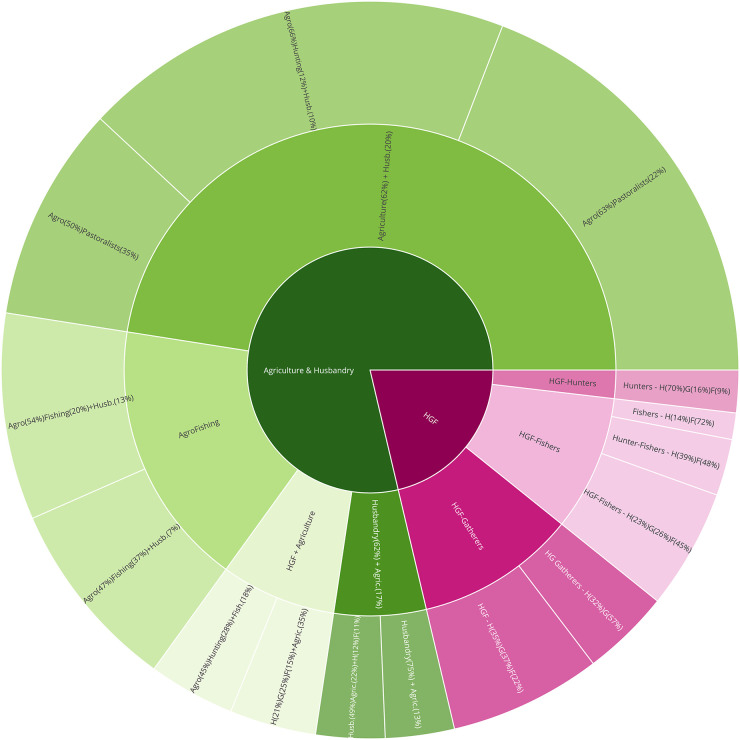
Sunburst of the different subsistence strategies identified via hierarchical clustering for *k* = 2, 7, 15. A short description is provided for each of the clusters. Note that the size of the circular sectors is proportional to the number of societies falling under each cluster.

In accordance with Fig 8 (the sunburst diagram), for *k* = 15 the *agrofishing* strategy is further divided into two clusters, and the same happens to the WSE. Consequently, in this section we will succinctly explore these four subdivisions, together with an additional cluster that is obtained after splitting the pastoralist cluster into two—recall that this fifth cluster will be also delved into since it happens to perfectly match the definition of mixed economies too. For the details on their geographical location and entropy values please refer to Fig 5 (map of the subsistence strategies with a relevant percentage of agriculture and/or husbandry for *k* = 15) and S6 Table (the summary of information entropies for *k* = 15) respectively.

### *Agrofishing* subdivisions

- **Cluster 6.A. (117/1290 societies): *Agro*(47%)*fishing* (37%) + Husbandry (7%).** In overall terms, this cluster could be described as the purely *agrofishing* strategy: the percentages of dependence on both agriculture and fishing are rather close, being the contribution of husbandry entirely marginal. Regarding their prevalent crop type, it is roots/tubers, followed by tree-fruits. The constituents of this cluster are mainly island societies living in tropical and subtropical moist broadleaf forests, except for those groups found in central Africa and in the external territories of the Amazon basin.
- **Cluster 6.B. (109/1290 societies): *Agro*(54%)*fishing*(20%) + Husbandry (13%).** This second *agrofishing* cluster corresponds to *agrofishing* inland societies; their subsistence economy is characterised by a predominance of cereal agriculture and a lower percentage of fishing that is compensated with a higher percentage of animal husbandry. The prevailing biome in which they appear is tropical and subtropical moist broadleaf forests.

### WSE subdivisions

- **Cluster 7.A. (49/1290 societies): *Agrohunting* + Fishing–Agriculture (45%) + Hunting (28%) + Fishing (18%).** This cluster presents a subsistence strategy in which plant resources are obtained from agriculture while animal products come from both hunting and fishing. Their crops consist of either cereals or roots/tubers with equal probability, and these societies are generally located in inland tropical and/or temperate ecosystems. One of their defining features is that they find themselves in the vicinity of lakes and big rivers. In this subsistence economy, the greater weight given to hunting with respect to fishing is likely to stem from their inland location—recall that even though lakes and big rivers provide exceptional fishing resources, their richness is not comparable to that of seas and/or oceans.
- **Cluster 7.B. (49/1290 societies): Paradigm of the Whole Spectra Economy–H(21%)G(25%)F(15%) + Agriculture (35%).** The characteristics of this WSE subdivision are very similar to those obtained for the WSE when *k* = 7. It is found across a wide range of different biomes, being their major crop type either cereals or roots/tubers with equal probability.

### Pastoralist mixed subdivision

- **Cluster 4.B. (39/1290 societies): Husbandry (49%) + Agriculture (22%) + Hunting (12%) + Fishing (11%).** This cluster corresponds to the second most mixed economy: the one with the second highest value of information entropy, only surpassed by that of the WSE (the paradigm of a mixed economy). Remarkably, this alternative is found in those biomes that are rather ill-suited for both agriculture and hunting: deserts and xeric shrublands and boreal forests, a fact that explains the great percentage of reliance on animal husbandry, as husbandry is famous for being highly resilient and successful in extremely adverse environments [191].

The conclusions to be drawn from the different clusters into which mixed economies are subdivided for *k* = 15 may be summarised as follows:

1. **Mixed Economies represent a complex phenomenon with multiple manifestations and significant internal variability.** When we defined *mixed economies*, we detailed their two distinctive characteristics, namely: (1) the combination of foraging and farming strategies within the same subsistence economy; and (2) the encompassing of a greater number of subsistence strategies (usually three or four). It is in the context of this second aspect that we find the greatest variability. An interesting way of looking at such variability is to arrange the different mixed economies along a gradient of increasing information entropy, or what is the same, increasing diversification. As it can be deduced from S6 Table, such a gradient would start with the *agrofishing* cluster 6.A.—which is admittedly a highly specialised alternative—and it would culminate with cluster 7.B., the paradigm of the Whole Spectra Economy—which constitutes the most diversified of all the subsistence strategies found. Therefore, from this point we can conclude that all the subsistence economies encompassed under *mixed economies* are indeed quite different from each other, a fact that is perfectly aligned with the notion that the middle ground was actually much wider, diverse and complex than previously thought.
2. **Diversification as a risk-management strategy.** In those mixed economies encompassing a greater number of subsistence strategies, and most notably in those with similar percentages of dependence on the different sources considered—i.e., the ones with the highest information entropy—diversification could in fact be a risk minimisation strategy, as complementarity allows to counterbalance possible shortages in specific resources [192,193]. Thereupon, the most diverse mixed economies would be the most flexible, resilient and reliable ones. Actually, risk and return maximisation appear to be managed differently in mixed vs. primary economies, since while mixed economies diversify, primary economies maximise return rates through specialisation, intensification and/or greater labour investment on a single subsistence strategy. This aspect is in all likelihood linked to their different socioecological contexts, as specialist strategies work well in stable environments, while diversification is more suitable for settings with unpredictable environmental events [194].
3. **The role of fishing in shaping mixed economies.** Fishing is present to a greater or lesser extent in all the economies that are mixed. Clearly, this point is very closely linked to the previous one, since as noted by Larson et al. [192], one of the major advantages of costal/riverine/lacustrine settlement is the ensuing reduction in the overall variance in food production, as a consequence of pooling the yields from different terrestrial and aquatic sources. Importantly, thanks to this risk-mitigation function, fishing could have been the key element around which mixed economies are structured, being at the same time responsible for their viability and long-term success. More precisely, as a result of the high carrying capacity of aquatic environments [187,195]—which translates into low risk and the easy attainment of greater returns by simply increasing labour investment and/or technological innovation—fishing could have acted as a buffer against adverse disturbances, hence enabling to face climatic downturns [128,196], population increases and/or competitive social dynamics (Weitzel et al 2020) without substantially changing the economic structure; that is, fishing would have conferred resilience to the socioeconomic system [197,198], and thus, prevented and/or delayed the adoption of alternative subsistence economies such as fully agricultural ones.

### Question 2: What is the role played by ecological settings in the configuration of the different subsistence strategies?

There has been much debate about the role played by ecological variables in shaping human behaviour—see Nettle et al. [199] and a review in Ahedo et al [200]. At present, one of the most widely accepted views is that even though they condition human agency, they do not determine it. In this line, despite human cultural variability might not be directly explained on the basis of ecological traits, it is true that ecological constraints shape subsistence activities [201], and that these configure several social organisational aspects.

Given our omnivorous nature, human nutritional requirements can be fulfilled in very different ways. Here is where the environmental conditions come into play, since while several ecological contexts allow the development of different and/or flexible economic strategies, many others offer limited possibilities, condition the complementarity choices to be made and restrain population growth. More specifically, whilst the carrying capacity of some settings can be further increased just through innovation, diversification, specialisation and/or intensification [202], in other contexts it is simply unfeasible to expand it (see for instance desertic areas in which agriculture cannot be put into practice). Nevertheless, it is important to note that not only the ecological variables set limits to growth; in fact, subsistence economies themselves present limitations as well, since they offer different growth possibilities depending on their structure. In HG societies with no food storage techniques, for instance, intensification is, to a major extent, limited, as increasing the hunting/gathering rates above the needs of the group would directly translate into those resources becoming spoiled [203,204]. By contrast, in fully agricultural societies with storage systems, a priori there would be no limitations to intensification other than those imposed by the land’s productivity and availability.

As far as our results are concerned, they show that specific socioeconomic choices appear recurrently in certain ecological systems. While this is obvious for coastal economies, our findings may also shed light into possible macro-behavioural patterns associated with human ecology, socioecological systems and their persistence. More specifically, in the cluster interpretation from question 1 (based on Figs 3 and 4) we found that (i) pastoralism and gathering are the prevailing subsistence strategies in arid areas; (ii) agriculture is extremely versatile, being viable in a wide range of biomes as long as they are warm and have water; (iii) the primacy of hunting appears recurrently in Subarctic contexts; (iv) those HGF whose dominant strategy is fishing are commonly found in cold coastal areas; (v) in *agrofishing* strategies, the different percentages of dependence on fishing resources correspond to their inland or coastal location; and that (vi) WSE more heavily reliant on hunting correspond to inland locations in the vicinity of lakes and/or rivers. Additionally, these findings are in perfect coherence with the results from the group variable importance analysis (Fig 6) which show that from all the groups of variables considered, the group of the ecological variables is the most discriminant one. Lastly, as regards the results from the individual variable importance analysis (Fig 5), it is worth noting that even though the variable biome itself contributes just marginally to predicting the response (when the predictors are arranged by importance, it appears in the last third), the fact is that many of the variables within the top half are to some extent related to the biome—see agriculture intensity, crop type, temperature and precipitation-related variables, resource richness, etc.

### Question 3: The role of fishing in the development of viable alternatives to cultivation

Archaeological and ethnographic narratives of the past 150 years have traditionally relegated fishing and aquatic-oriented societies to a marginal role; the reasons behind such relegation are to be found (among others) in (i) the postglacial sea-level rise, which submerged most of the shorelines older than 10,000 years B.P. and thus their archaeological evidence, a fact that has led to assumption that aquatic resource exploitation emerged very late in human prehistory; (ii) the differential preservation and reporting of material evidence of aquatic adaptations (such evidence is very scanty as most tools were made from biodegradable materials, and its documentation is considerably patchy since aquatic resources were held to be economically unimportant); (iii) all the additional biases of the archaeological and ethnographic records; (iv) the predominant role attributed to hunting in most traditional hunter-gatherer models; and (v) the reluctance to abandon the deep-seated HG-farmer classification and the ladder of economic and technological progress it implies [129,195].

However, the results of our analyses show that the role of fishing could have been far more relevant than previously thought. In fact, in all but the purest primary economies, the percentage of dependence on fishing is greater than 10%. For the resolution level corresponding to *k* = 7, we see that fishing constitutes a relevant source of food in 4 out of 7 (60%) of the clusters identified, being its contribution only marginal in clusters 2 (hunters), 4 (pastoralists) and 5 (agriculturalists). This 60% ratio is maintained for *k* = 15, where the percentage of dependence on fishing is found to be either equal to or greater than 10% in 9 out of 15 of the clusters. In this vein, it is also remarkable that with the exception of the strictly fishing economies (i.e., the HGF heavily reliant on fishing and the sheer fishers), the presence of fishing is a common factor across all those subsistence economies that diversify more, that is, that are more ‘mixed’. As a matter of fact, if we look at the clusters obtained for *k* = 15 (please refer to S6 Table in the Supplementary Material) from cluster 6.A. (*agrofishing*) onwards, we identify both a gradient of increasingly mixed subsistence economies, and the presence of a relevant percentage of fishing across all them. Therefore, we can assert that fishing is the common denominator of all the strategies lying between *agrofishing* and the WSE. Actually, *agrofishing* seems to play a ‘hinge’ role between the primary economies (i.e., those heavily reliant on one to two subsistence strategies and that generally correspond to the traditional stereotypes of HFG, pastoralism, agriculture and/or agropastoralism), and the mixed economies, the most paradigmatic of which is the WSE. Recall that this finding is aligned with previous contributions by Erlandson et al. [205,206] and Lepofsky et al. [196], in which they already noted that in accordance with recent findings, our ancestors would have relied on aquatic resources more heavily and for longer periods of time than the twentieth century anthropological theory once suggested.

As previously mentioned, aquatic environments (coastal, riverine and/or lacustrine) constitute exceptionally rich ecosystems [195] in which—at least a priori—neither resource restoration nor limits of carrying capacity pose a problem, since both can generally be solved by simply enlarging and/or changing the catchment area. In these contexts, the main limitations are linked either to geographical constraints and/or to the development of specific nautical/resource-exploitation technologies. Due to all the above, aquatic environments can be considered singular locations that present very specific subsistence behaviours and that foster particular social dynamics.

Regarding subsistence economies, aquatic societies are known to have developed sophisticated management systems to sustain or increase resource diversity and/or yields. The broad spectrum of activities, actions and strategies encompassed under management systems may be organised around four main pillars: (i) harvesting methods (see for instance clam harvesting with digging sticks, *en masse* fish harvesting by means of weirs, traps and nets, the extension of harvest times through the construction of holding ponds into intertidal fish traps, the establishment of harvesting rules to prevent overharvesting, etc.) [196]; (ii) enhancement strategies (such as transplanting eggs, size selection, habitat conditioning and extension: boulder clearance, construction of rock walls in the lowest intertidal zone to create clam gardens, etc.) [126,207]; (iii) tenure systems (ownership of fish harvesting locations and/or rights to catch fish from certain areas) [208]; and (iv) world view and social realm (discouragement of overharvesting, pursuit of ecosystem equilibrium, initiation ceremonies) [128]. In the ethnographic and archaeological records, paradigmatic examples of aquatic management techniques are documented—among others—for the Chulmun people in Korea [123], several coastal societies from the Atacama Desert [209] and indigenous peoples from both the NW Coast of North America [210] and the Southern Coasts of Eastern North America [211].

As far as social dynamics are concerned, aquatic-oriented societies are typically characterised by their reduced mobility [212,213] (which in most cases would have led to sedentarism or semi-sedentarism [214]), their big population numbers [111,196], and/or the emergence of social organisational changes such as higher territoriality [215] and/or increased political complexity [216,217], traits long assumed to require agriculture to develop.

Eventually, we would like to return to the fact that as a consequence of their high resilience, successful aquatic/maritime adaptations could have acted as a viable alternative to cultivation [41]. Coastal/riverine/lacustrine societies would have retained their hunter-gatherer strategies or would have adopted different subsistence combinations strongly reliant on aquatic resources, since fishing would have been significantly more reliable and cost-efficient than other inland alternatives [218,219]. In this regard, it is also remarkable that the adoption of subsistence economies significantly dependent on fishing would not have been the immediate consequence of living in a coastal/aquatic environment; quite the contrary, it would have been the result of a thoughtful choice after carefully considering the risks and advantages of other alternatives; for instance, several examples attest to the retention of the original subsistence economies after having continuous contact with farming populations [130,219,220].

## Conclusions

Current research on the transition to agriculture is characterised by both a burgeoning corpus of data and the pursuit of broader explanatory frameworks through the integration of different theoretical perspectives and the adoption of computational approaches. In this context, the present contribution proposes to explore prehistoric economies in general, and the middle ground in particular, through the quantitative analysis of ethnographically documented subsistence choices. More specifically, by means of both unsupervised and supervised learning techniques, we have assessed (i) the viability and relevance of the different subsistence combinations, (ii) the existence of associations between specific subsistence economies and certain ecological settings, and (iii) the importance of fishing in the configuration of mixed economic choices. The main conclusions obtained may be summarised as follows:

1. Recurrent specific subsistence combinations have been identified via clustering in the EA subsistence dataset, which implies that not all combinations are viable. For the different levels of resolution considered, the subsistence economies found can be divided into two main groups: *primary economies* and *mixed economies*. Even though such divide may not seem innovative (since those designations have already been used in the literature), the fact is that in contrast to all the previous contributions (most of which consist in case-based theorisation), we propose a formal quantitative criteria (the information entropy) to determine whether a subsistence economy is mixed: the higher the information entropy value, the more mixed the subsistence economy.
2. Mixed economies are not a marginal choice. In fact, they represent up to a quarter of the cases in our EA subsistence dataset. In addition, the *mixed economies* group presents clear patterns of internal variability that are directly correlated with the diversification level of the different alternatives. Remarkably, such diversification may actually be a risk-management strategy that could be responsible for the high resilience characteristic of mixed economies.
3. Fishing as a subsistence strategy is more relevant than previously thought, being present in up to 60% of the different subsistence economies identified. In addition, fishing seems to be the key element around which mixed economies are structured, as it is the common denominator across all of them. Notably, given that aquatic environments are characterised by their high resource richness, fishing could have acted as a buffer against social and/or environmental contingencies, thus ensuring the long-term persistence of all the strategies of which it is part, and favouring their consolidation as viable alternatives to cultivation.

Ultimately, our contribution serves to illustrate the potential of advanced computational methods as theory-building tools; more precisely, we have showed that the application of machine learning techniques to a comprehensive standardised cross-cultural database such as D-PLACE can provide us with new insights into problems as relevant, complex and multidimensional as the transition to agriculture.

## Supporting information

S1 Fig Ridgeline plot of the entropy distributions of each cluster for k = 2. Recall that the distributions have been sorted in ascending order of entropy along the vertical axis.
(EPS)

S2 Fig Ridgeline plot of the entropy distributions of each cluster for *k* = 15. Recall that the distributions have been sorted in ascending order of entropy along the vertical axis.
(EPS)

S1 Table Succinct summary of the main theories on the Origins of Agriculture described in the manuscript and the most relevant references. (DOCX)

S2 Table Correlation coefficients and their respective *p*-values (in brackets) of the first PCA dimensions with the variables in the dataset: Percentage of dependence on hunting, gathering, fishing, husbandry and agriculture. (DOCX)

S3 Table Optimal number of clusters proposed by elbow method, average silhouette method, gap statistic and NbClust following the majority rule. (DOCX)

S4 Table Most frequent alternatives proposed as the optimal number of clusters by the 30 different indices computed by NbClust. Note that this table is some sort of contingency table and that proposals receiving just one vote have not been included.
(DOCX)

S5 Table Summary table for *k* = 2. It includes cluster number, each cluster’s average strategy, -the average of the percentages of dependence on gathering, hunting, fishing, husbandry and agriculture across all societies in the cluster-, their entropy, standard deviation, the number of variables with a percentage of dependence equal or greater than 15% and 10%, and a succinct interpretation of the cluster. Note that the table has been sorted in ascending order of entropy.
(DOCX)

S6 Table Summary table for *k* = 15. It includes cluster number, each cluster’s average strategy, -the average of the percentages of dependence on gathering, hunting, fishing, husbandry and agriculture across all societies in the cluster-, their entropy, standard deviation, the number of variables with a percentage of dependence equal or greater than 15% and 10%, and a succinct interpretation of the cluster. Note that the table has been sorted in ascending order of entropy.
(DOCX)

S1 Appendix Choice of *k* = 7 and *k* = 15. (DOCX)
