# Supplementary material for: Let’s go fishing: A quantitative analysis of subsistence choices with a special focus on mixed economies among small-scale societies
Source: PLoS One. 2021 Aug 4;16(8):e0254539. doi: 10.1371/journal.pone.0254539 (PMC8336859; doi:10.1371/journal.pone.0254539)
Supplement: S1 Table — (DOCX) [file pone.0254539.s003.docx]

| **Explanatory framework** | **Theoretical proposals** | **References** |
| --- | --- | --- |
| Traditional universalist prime-mover explanations | Agriculture as the superior mode of production | [10] [11–15] |
|  | SET-based explanations for initial domestication | [31], [32,33], [34,35]  [36,37], [38–41]  [42–49], [32,50–52] |
|  | Social hypothesis | [30,53–56], [19] |
| Recent alternatives and integrative approaches | Approaches derived from Evolutionary Ecology (EE) and Human Behavioural Ecology (HBE) | [4,58–60], [71]  [4,69,72–74], [63] |
|  | Niche-Construction-Theory-(NCT)-based approaches | [2], [9,61,62,77,78] |
|  | Integrative approaches comprising EE, NCT and models of cultural transmission and gene-culture coevolution | [58,63–69], [3,6,8,228] |

Table S 1. Succinct summary of the main theories on the Origins of Agriculture described in the manuscript and the most relevant references
