# Supplementary material for: Let’s go fishing: A quantitative analysis of subsistence choices with a special focus on mixed economies among small-scale societies
Source: PLoS One. 2021 Aug 4;16(8):e0254539. doi: 10.1371/journal.pone.0254539 (PMC8336859; doi:10.1371/journal.pone.0254539)
Supplement: S2 Table — (DOCX) [file pone.0254539.s004.docx]

| **PC Dim.** | **Hunting** | **Gathering** | **Fishing** | **Husbandry** | **Agriculture** |
| --- | --- | --- | --- | --- | --- |
| 1 | 0.806 (2.319e-295) | 0.775 (5.286e-259) | 0.551 (2.555e-103) | -0.593 (2.167e-123) | -0.848 (0.000e+00) |
| 2 | 0.245 (4.834e-19) | 0.346 (1.130e-37) | -0.670 (4.802e-169) | 0.575 (3.079e-114) | -0.288 (3.905e-26) |
| 3 | -0.175 (2.579e-10) | -0.225 (2.525e-16) | 0.495 (1.561e-80) | 0.564 (4.256e-109) | -0.445 (7.917e-64) |
| 4 | 0.510 (2.154e-86) | -0.478 (1.256e-74) | - | - | - |

Table S 2. Correlation coefficients and their respective *p*-values (in brackets) of the first PCA dimensions with the variables in the dataset: percentage of dependence on hunting, gathering, fishing, husbandry and agriculture.
