# Supplementary material for: Let’s go fishing: A quantitative analysis of subsistence choices with a special focus on mixed economies among small-scale societies
Source: PLoS One. 2021 Aug 4;16(8):e0254539. doi: 10.1371/journal.pone.0254539 (PMC8336859; doi:10.1371/journal.pone.0254539)
Supplement: S3 Table — (DOCX) [file pone.0254539.s005.docx]

| **Method** | **Optimal nb. of clusters** |
| --- | --- |
| Elbow method | 2 |
| Average silhouette method | 2 |
| Gap statistic | 9 |
| NbClust (majority rule) | 2 |

Table S 3. Optimal number of clusters proposed by elbow method, average silhouette method, gap statistic and NbClust following the majority rule.
