# Supplementary material for: Let’s go fishing: A quantitative analysis of subsistence choices with a special focus on mixed economies among small-scale societies
Source: PLoS One. 2021 Aug 4;16(8):e0254539. doi: 10.1371/journal.pone.0254539 (PMC8336859; doi:10.1371/journal.pone.0254539)
Supplement: S4 Table — Note that this table is some sort of contingency table and that proposals receiving just one vote have not been included. (DOCX) [file pone.0254539.s006.docx]

| **Optimal nb. of clusters** | **Nb. of NbClust indices proposing it** |
| --- | --- |
| 2 | 6 |
| 3 | 5 |
| 4 | 2 |
| 5 | 4 |
| 15 | 3 |

Table S 4. Most frequent alternatives proposed as the optimal number of clusters by the 30 different indices computed by NbClust. Note that this table is some sort of contingency table and that proposals receiving just one vote have not been included.
