# Supplementary material for: Let’s go fishing: A quantitative analysis of subsistence choices with a special focus on mixed economies among small-scale societies
Source: PLoS One. 2021 Aug 4;16(8):e0254539. doi: 10.1371/journal.pone.0254539 (PMC8336859; doi:10.1371/journal.pone.0254539)
Supplement: S5 Table — It includes cluster number, each cluster’s average strategy, -the average of the percentages of dependence on gathering, hunting, fishing, husbandry and agriculture across all societies in the cluster-, their entropy, standard deviation, the number of variables with a percentage of dependence equal or greater than 15% and 10%, and a succinct interpretation of the cluster. Note that the table has been sorted in ascending order of entropy. (DOCX) [file pone.0254539.s007.docx]

|  | **Clusters’ average strategies (Mean values per variable and cluster)** | | | | |  |  |  |  |  |
| --- | --- | --- | --- | --- | --- | --- | --- | --- | --- | --- |
| **Cluster nb** | **Gathering (%)** | **Hunting (%)** | **Fishing (%)** | **Husbandry (%)** | **Agriculture (%)** | **Entropy** | **SD** | **Limit 15** | **Limit 10** | **Interpretation** |
| 2 | 5,73 | 9,43 | 11,96 | 19,29 | 53,58 | 1,29 | 19,42 | 2 | 3 | Agriculture & Husbandry |
| 1 | 30,78 | 33,75 | 29,32 | 2,68 | 3,47 | 1,30 | 15,54 | 3 | 3 | HGF |

Table S 5. Summary table for *k* = 2. It includes cluster number, each cluster’s average strategy, -the average of the percentages of dependence on gathering, hunting, fishing, husbandry and agriculture across all societies in the cluster-, their entropy, standard deviation, the number of variables with a percentage of dependence equal or greater than 15% and 10%, and a succinct interpretation of the cluster. Note that the table has been sorted in ascending order of entropy.
