# Supplementary material for: Let’s go fishing: A quantitative analysis of subsistence choices with a special focus on mixed economies among small-scale societies
Source: PLoS One. 2021 Aug 4;16(8):e0254539. doi: 10.1371/journal.pone.0254539 (PMC8336859; doi:10.1371/journal.pone.0254539)
Supplement: S6 Table — It includes cluster number, each cluster’s average strategy, -the average of the percentages of dependence on gathering, hunting, fishing, husbandry and agriculture across all societies in the cluster-, their entropy, standard deviation, the number of variables with a percentage of dependence equal or greater than 15% and 10%, and a succinct interpretation of the cluster. Note that the table has been sorted in ascending order of entropy. (DOCX) [file pone.0254539.s008.docx]

|  | **Clusters’ average strategies (Mean values per variable and cluster)** | | | | |  |  |  |  |  |
| --- | --- | --- | --- | --- | --- | --- | --- | --- | --- | --- |
| **Cluster nb** | **Gathering (%)** | **Hunting (%)** | **Fishing (%)** | **Husbandry (%)** | **Agriculture (%)** | **Entropy** | **SD** | **Limit 15** | **Limit 10** | **Interpretation** |
| 4.A. | 2,88 | 6,58 | 2,90 | 75,02 | 12,62 | 0,86 | 31,01 | 1 | 2 | Husbandry (75%) + Agric. (13%) |
| 2.BIS. | 16,40 | 69,82 | 8,50 | 2,64 | 2,64 | 0,95 | 28,42 | 2 | 2 | Hunters - H(70%)G(16%)F(9%) |
| 3.A. | 5,32 | 13,85 | 71,65 | 4,28 | 4,90 | 0,95 | 29,14 | 1 | 2 | Fishers - H(14%)F(72%) |
| 1.A. | 56,58 | 31,60 | 5,20 | 2,64 | 3,99 | 1,06 | 23,72 | 2 | 2 | HG Gatherers - H(32%)G(57%) |
| 5.A. | 4,23 | 5,22 | 5,84 | 21,98 | 62,73 | 1,08 | 24,99 | 2 | 2 | Agro(63%)Pastoralists(22%) |
| 3.B. | 7,97 | 39,03 | 48,09 | 2,57 | 2,34 | 1,10 | 21,86 | 2 | 2 | Hunter-Fishers - H(39%)F(48%) |
| 5.C. | 5,89 | 11,59 | 6,93 | 9,72 | 65,87 | 1,10 | 25,74 | 1 | 2 | Agro(66%)Hunting(12%) + Husb.(10%) |
| 5.B. | 3,47 | 7,52 | 4,03 | 34,88 | 50,11 | 1,15 | 21,28 | 2 | 2 | Agro(50%)Pastoralists(35%) |
| 6.A. | 3,75 | 5,67 | 36,72 | 7,28 | 46,58 | 1,20 | 20,11 | 2 | 2 | Agro(47%)Fishing(37%) + Husb.(7%) |
| 3.C. | 25,78 | 23,16 | 45,14 | 2,47 | 3,45 | 1,25 | 17,73 | 3 | 3 | HGF - Fishers - H(23%)G(26%)F(45%) |
| 6.B. | 5,73 | 6,62 | 20,27 | 13,11 | 54,29 | 1,26 | 20,04 | 2 | 3 | Agro(54%)Fishing(20%) + Husb.(13%) |
| 1.B. | 36,68 | 34,82 | 22,28 | 2,64 | 3,58 | 1,28 | 16,39 | 3 | 3 | HGF - H(35%)G(37%)F(22%) |
| 7.A. | 5,50 | 28,12 | 17,91 | 3,93 | 44,54 | 1,31 | 16,89 | 3 | 3 | Agro(45%)Hunting(28%) + Fish.(18%) |
| 4.B. | 5,99 | 12,43 | 10,65 | 48,75 | 22,18 | 1,35 | 17,12 | 2 | 4 | Husbandry (49%) + Agric. (22%) + H(12%)F(11%) |
| 7.B. | 24,91 | 20,98 | 15,01 | 3,66 | 35,44 | 1,45 | 11,78 | 4 | 4 | H(21%)G(25%)F(15%) + Agric.(35%) |

Table S 6. Summary table for *k* = 15. It includes cluster number, each cluster’s average strategy, -the average of the percentages of dependence on gathering, hunting, fishing, husbandry and agriculture across all societies in the cluster-, their entropy, standard deviation, the number of variables with a percentage of dependence equal or greater than 15% and 10%, and a succinct interpretation of the cluster. Note that the table has been sorted in ascending order of entropy.
