# Supplementary material for: Let’s go fishing: A quantitative analysis of subsistence choices with a special focus on mixed economies among small-scale societies
Source: PLoS One. 2021 Aug 4;16(8):e0254539. doi: 10.1371/journal.pone.0254539 (PMC8336859; doi:10.1371/journal.pone.0254539)
Supplement: S1 Appendix — (DOCX) [file pone.0254539.s009.docx]

S1 Appendix – Choice of *k* = 7 and *k* = 15.


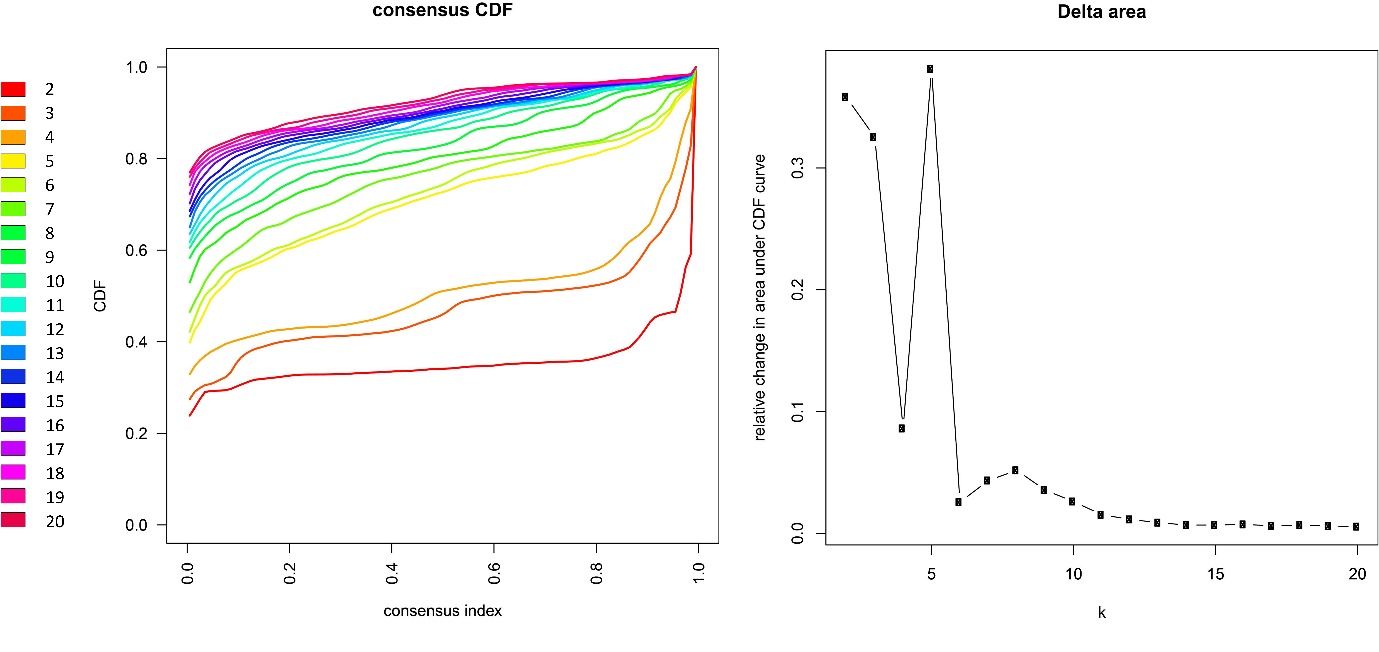


Fig S 1. Cumulative distribution functions (CDFs) obtained for the different *k’*s (left), and delta area function (right), which shows the increase in the area under the CDF with increasing *k*.

As stated in the Results section, the assessment of the evolution of the CDFs and of the delta function -both showed in Figure S 1- lead us to the identification of two levels of interest: (i) the one ranging from *k* = 5 to *k* = 10 and (ii) that ranging from *k* = 11 up to *k* = 20.

As far as level (i) is concerned, at *k* = 5 we find the steepest increase in the area under the CDF; the ensuing *k’*s up to *k* = 10 do also translate into an increase in the area under the CDF, but of a significantly smaller magnitude. Therefore, we opted for *k* = 7 to explore level (i) since even though in accordance with the delta area one would probably choose *k* = 8, the consensus matrix obtained for *k* = 7 is undoubtedly more diagonal-block and cleaner -hence denoting more cluster stability- that the one for *k* = 8. To see the consensus matrices obtained for *k* = 2 to *k* = 20 please refer to the folder ***Consensus matrices & CDF* available at our GitHub repository:** <https://github.com/Virahe/Lets-go-Fishing>.

Regarding level (ii), it seems to saturate after *k* = 13, being the increases in the area under the CDF after that value indeed very small; however, we chose the intermediate value of the interval, i.e., *k* = 15, since again even though according to the delta function one would probably choose *k* = 13, the consensus matrix for *k* = 15 is in fact more diagonal-block and consequently the cluster structure is likely to be more stable.
